# Alanine Scanning Mutagenesis of the C-Terminal Cytosolic End of Gpm6a Identifies Key Residues Essential for the Formation of Filopodia

**DOI:** 10.3389/fnmol.2018.00314

**Published:** 2018-09-04

**Authors:** Nicolás M. Rosas, Anabel Alvarez Juliá, Sofia E. Alzuri, Alberto C. Frasch, Beata Fuchsova

**Affiliations:** Instituto de Investigaciones Biotecnológicas IIB-INTECH, CONICET-UNSAM, San Martin, Argentina

**Keywords:** filopodium, primary hippocampal neuron, neuroblastoma cells N2a, mutagenesis, C-terminal cytosolic end, membrane glycoprotein M6a

## Abstract

Neuronal membrane glycoprotein M6a (Gpm6a) is a protein with four transmembrane regions and the N- and the C-ends facing the cytosol. It functions in processes of neuronal development, outgrowth of neurites, and formation of filopodia, spines, and synapsis. Molecular mechanisms by which Gpm6a acts in these processes are not fully comprehended. Structural similarities of Gpm6a with tetraspanins led us to hypothesize that, similarly to tetraspanins, the cytoplasmic tails function as connections with cytoskeletal and/or signaling proteins. Here, we demonstrate that the C- but not the N-terminal cytosolic end of Gpm6a is required for the formation of filopodia by Gpm6a in cultured neurons from rat hippocampus and in neuroblastoma cells N2a. Further immunofluorescence microcopy and flow cytometry analysis show that deletion of neither the N- nor the C-terminal intracellular domains interferes with the recognition of Gpm6a by the function-blocking antibody directed against the extracellular part of Gpm6a. Expression levels of both truncation mutants were not affected but we observed decrease in the amount of both truncated proteins on cell surface suggesting that the incapacity of the Gpm6a lacking C-terminus to induce filopodium formation is not due to the lower amount of Gpm6a on cell surface. Following colocalization assays shows that deletion of the C- but not the N-terminus diminishes the association of Gpm6a with clathrin implying involvement of clathrin-mediated trafficking events. Next, using comprehensive alanine scanning mutagenesis of the C-terminus we identify K250, K255, and E258 as the key residues for the formation of filopodia by Gpm6a. Substitution of these charged residues with alanine also diminishes the amount of Gpm6a on cell surface and in case of K255 and E258 leads to the lower amount of total expressed protein. Subsequent bioinformatic analysis of Gpm6a amino acid sequence reveals that highly conserved and functional residues cluster preferentially within the C- and not within the N-terminus and that K250, K255, and E258 are predicted as part of sorting signals of transmembrane proteins. Altogether, our results provide evidence that filopodium outgrowth induced by Gpm6a requires functionally critical residues within the C-terminal cytoplasmic tail.

## Introduction

TheGpm6abelongs to the proteolipid protein (Plp/Dm20) gene family and is abundantly expressed in neurons of the central nervous system (CNS) (Yan et al., 1993, 1996). Pathological conditions have been linked to the alterations in *GPM6A* expression levels or sequence. Downregulation of *GPM6A* mRNA levels has been shown in the hippocampus of depressed suicide victims (Fuchsova et al., 2015) and the association of *GPM6A* gene with schizophrenia (Boks et al., 2008; Ma et al., 2018), bipolar disorders (Greenwood et al., 2012), and claustrophobia (El-Kordi et al., 2013) has been described. On the other hand, *de novo* duplication of *GPM6A* gene leading to the higher expression of *GPM6A* has been connected to learning disability and anomalies in the behavior (Gregor et al., 2014) suggesting the importance of accurate expression of *GPM6A* for cognitive function. In several animal models, chronic stress, an agent critically involved in the etiology of depression, alters expression levels of Gpm6a and this effect is counteracted by treatment with antidepressants (Alfonso et al., 2004a,b; Cooper et al., 2009; Monteleone et al., 2014).

The roles of Gpm6a in the nervous system are incompletely comprehended. However, there is abundant evidence for its participation in filopodium formation, neurite extension, synaptogenesis (Lagenaur et al., 1992; Mukobata et al., 2002; Alfonso et al., 2005; Michibata et al., 2008; Zhao et al., 2008; Fuchsova et al., 2009; Brocco et al., 2010; Huang et al., 2011; Scorticati et al., 2011; Formoso et al., 2015; Mita et al., 2015), neuronal differentiation of human stem cells (Michibata et al., 2008) and PC12 cells (Mukobata et al., 2002), as well as in determination of neuronal polarity during neurite formation in neuronal development (Honda et al., 2017; Ito et al., 2018). In addition, Gpm6a has been shown to interact with the micro-opioid receptor [and with a number of other G protein-coupled receptors (GPCRs)] and to facilitate receptor endocytosis and recycling (Wu et al., 2007; Liang et al., 2008). Endocytic sorting and recycling of Gpm6a involves clathrin-dependent pathway and affects neuronal synapses (Garcia et al., 2017).

Overexpression of Gpm6a in rat hippocampal neurons or in cells of neuronal (N2a, PC12) as well as non-neuronal (COS7) origin leads to the vast formation of filopodia, while decrease of endogenous Gpm6a expression by siRNA reduces filopodium density (Alfonso et al., 2005). Filopodia are slender protrusions of plasma membrane filled with actin filaments that underlie many major morphogenetic events in the nervous system (Mattila and Lappalainen, 2008; Gallo, 2013). They are required to initiate extension of neurites and their ramification (Dent et al., 2007). They are also present in neuronal growth cones where they guide axons and dendrites (Gallo and Letourneau, 2004), and filopodia in dendrites function as precursors of spines (Sekino et al., 2007) that create postsynaptic regions of most excitatory synapses.

The mechanism that mediates formation of filopodia by Gpm6a is not fully understood. The localization of Gpm6a in lipid microdomains as well as Src kinases and MAPK activity have been reported to participate (Scorticati et al., 2011). More recently, it has been shown that the actin regulator Coronin 1a associates with Gpm6a in rat hippocampal neurons and facilitates the formation of filopodia by Gpm6a and Rac1/Pak1 signaling pathway has been shown to be involved (Fuchsova et al., 2015; Alvarez Julia et al., 2016).

In the present study our objective was to determine the regions of Gpm6a molecule that are required for the formation of filopodia. To identify the putative functionally important amino acid residues we took advantage of the striking structural similarities that Gpm6a shares with tetraspanin family of proteins: four transmembrane domains (TM1-4), two extracellular loops (EC1 and EC2), small intracellular loop (IC), and the N- and C-terminal regions facing the cytoplasm (**Supplementary Table S1**). Biological function of tetraspanins is facilitated by their capacity to interact with a number of proteins and this way regulate their spatial juxtaposition on the plasma membrane leading to co-ordination of signaling pathways (Hemler, 2005; Charrin et al., 2009; Yanez-Mo et al., 2009). In tetraspanins, functional specificity is determined by EC2 region and the cytoplasmic tail regions function as connections with cytoskeletal or signaling proteins. Similarly to tetraspanins, EC2 region of Gpm6a contains cysteine residues involved in the formation of disulfide bridges that are important for the topology of the domain and critical for the role of the protein in filopodium outgrowth (Fuchsova et al., 2009). In like manner, we hypothesized that the N- and C-terminal cytoplasmic tails of Gpm6a may be functionally crucial sites that mediate cross-talks with intracellular signaling and/or cytoskeletal structures required in the process.

In this report, we show that the C-terminal but not the N-terminal cytosolic end of Gpm6a is critical for the process of Gpm6a-induced filopodium formation. Subsequent alanine scanning mutagenesis of the C-terminal cytosolic end identifies K250, K255, and E258 as the key residues. Bioinformatic analysis of Gpm6a amino acid sequence reveals that the residues K250, K255, and E258 are predicted as part of signals for sorting of transmembrane proteins. Moreover, our colocalization assays show that deletion of the C- but not the N-terminal cytosolic domain diminishes the association of Gpm6a with clathrin implying involvement of clathrin-mediated trafficking events. Altogether, our results provide evidence that filopodium outgrowth induced by Gpm6a requires functionally critical residues within the C-terminal cytoplasmic tail. We propose that these residues could participate in the interaction of the C-terminal end of Gpm6a with other proteins that could directly regulate actin cytoskeleton dynamics or cell signaling, thus, facilitating the formation of filopodia or, alternatively, they could form part of post-translational modifications or structural motifs involved in the process.

## Materials and Methods

### Materials

Mammalian expression plasmids: pEGFP-C1 (Clontech) encoding the EGFP, EGFP-tagged wildtype (wt) full length mouse Gpm6a (Gpm6a wt-EGFP) described previously (Fuchsova et al., 2009). Primary antibodies: monoclonal anti-Gpm6a rat IgG (1/250; Medical and Biological Laboratories), rabbit anti-GFP polyclonal serum (1/1000; Thermo Fisher Scientific), monoclonal anti-alpha-tubulin mouse IgG1 (1/2000, Sigma), rabbit anti-clathrin heavy chain polyclonal antibody (1/400; Cell Signaling Technology). Secondary antibodies: rhodamine red-conjugated goat anti-rat IgG (1/1000; Jackson), goat anti-rat IgG conjugated to Alexa Fluor (AF) 647 (1/500, BioLegend), goat anti-rabbit conjugated to AF 568 (1/1000, Thermo Fisher Scientific).

### Site-Directed Mutagenesis

Mutations were introduced into the Gpm6a wt-EGFP, a mammalian expression plasmid that codes for the full length wt mouse Gpm6a (NCBI Accession: NP_705809.1) fused N-terminally with EGFP (vector pEGFP-C1; Clontech) that has been described previously (Fuchsova et al., 2009). The expressed fusion protein is functional and localizes correctly to the plasma membrane of transfected cell lines or neuronal primary cultures. To create deletion mutants lacking the N-terminal (ΔN) or the C-terminal (ΔC) intracellular domains, amino acids (aa) 1–16 or aa 243–278, respectively, were deleted. For alanine scanning mutagenesis done as described by Gibbs and coworkers (Gibbs and Zoller, 1991), charged amino acids of the C-terminal cytosolic end (aa 241–278) of the mouse Gpm6a at positions 243, 244, 247, 250, 252, 253, 255, 257, 258, 259, 261, 263, 264, 266, 269, 271, 272, 273 were substituted with alanine (**Figure 4A**). To keep the number of mutants to manageable proportions, two or three charged residues were mutated simultaneously when they occured together within a cluster of six or seven residues (Gibbs and Zoller, 1991). Nonconserved charged residues were not mutated in conjunction with charged residues identified as universally conserved in homologous proteins from the same family (**Supplementary Figure S1**) since highly conserved residues often play important functional or structural roles and functional information on any less conserved residue mutated simultaneously could be lost. Mutations were generated by a standard PCR technique using a *Pfu* DNA polymerase (Promega). Two overlapping oligonucleotide primers (Macrogen) with the forward or both primers containing the target mutation were used to amplify the template DNA and were as follows: **ΔN (Δ1-16)** 5′-TCT GCA GTC GAC GGT ACC TGC TGC ATT AAA TGC C-3′, reverse 5′-GGT ACC GTC GAC TGC AGA ATT CGA AGC TTG AGC TC-3′; **ΔC (Δ243–278)** 5′-AAC TGG GCC TAT GTG TAA GGG CCC TGC CGC ATG CA-3′, reverse 5′-CAC ATA GGC CCA GTT GGC AGA CAG AAC CAT CAG GTA G-3′; **K243A** 5′-AAC TGG GCC TAT GTG GCT GAT GCC TGC CGC ATG CAG AAG-3′, reverse 5′-CAC ATA GGC CCA GTT GGC AGA CAG AAC CAT CAG GTA G-3′; **D244A** 5′-CC TAT GTG AAA GCT GCC TGC CGC ATG CAG-3′, reverse 5′-TTT CAC ATA GGC CCA GTT GGC AGA CAG AAC CAT C-3′; **R247A** 5′-TAT GTG AAA GAT GCC TGC GCT ATG CAG AAG TAC GAA G-3′, reverse 5′-GCA GGC ATC TTT CAC ATA GGC CCA GTT GG-3′; **K250A** 5′-GCC TGC CGC ATG CAG GCT TAC GAA GAC ATC AAG TC-3′, reverse 5′-CTG CAT GCG GCA GGC ATC TTT CAC ATA GGC-3′; **E252A** 5′-CGC ATG CAG AAG TAC GCT GAC ATC AAG TCA AAG G-3′, reverse 5′-GTA CTT CTG CAT GCG GCA GGC ATC TTT CAC ATA GG-3′; **D253A/K255A** 5′-TGC CGC ATG CAG AAG TAC GAA GCC ATC GCG TCA AAG GAA GAG-3′, reverse 5′-CTC TTC CTT TGA CGC GAT GGC TTC GTA CTT CTG CAT GCG GCA-3′; **D253A** forward 5′-ATG CAG AAG TAC GAA GCT ATC AAG TCA AAG GAA GAG CAG-3′, reverse 5′-TTC GTA CTT CTG CAT GCG GCA GGC ATC TTT C-3′; **K255A** forward 5′-ATG CAG AAG TAC GAA GAC ATC GCT TCA AAG GAA GAG CAG-3′, reverse 5′-CTG CTC TTC CTT TGA AGC GAT GTC TTC GTA CTT CTG CAT-3′; **K257A** 5′-G AAG TAC GAA GAC ATC AAG TCA GCG GAA GAG CAG GAG-3′, reverse 5′-CTC CTG CTC TTC CGC TGA CTT GAT GTC TTC GTA CTT C-3′; **E258A/E259A** 5′-GAC ATC AAG TCA AAG GCT GCT CAG GAG CTG CAC-3′, reverse 5′-CTT TGA CTT GAT GTC TTC GTA CTT CTG CAT GCG GCA GGC ATC-3′; **E258A** forward 5′-GAC ATC AAG TCA AAG GCT GAG CAG GAG CTG CAC-3′, reverse 5′-GTG CAG CTC CTG CTC AGC CTT TGA CTT GAT GTC-3′; **E259A** forward 5′-GAC ATC AAG TCA AAG GAA GCT CAG GAG CTG CAC-3′, reverse 5′-CTT TGA CTT GAT GTC TTC GTA CTT CTG CAT GCG GCA GGC ATC-3′; **E261A/D264A** 5′-TCA AAG GAA GAG CAG GCT CTG CAC GCT ATC CAT TCT ACT C-3′, reverse 5′-CTG CTC TTC CTT TGA CTT GAT GTC TTC GTA CTT CTG C-3′; **H263A** forward 5′-TCA AAG GAA GAG CAG GAG CTG GCT GAC ATC CAT TCT ACT C-3′, reverse 5′-CAG CTC CTG CTC TTC CTT TGA CTT GAT GTC TTC GTA CTT CTG C-3′; **H266A/R269A** forward 5′-GAG CTG CAC GAC ATC GCT TCT ACT GCT TCC AAA GAG C-3′, reverse 5′-GAT GTC GTG CAG CTC CTG CTC TTC CTT TGA CTT GAT GTC TTC G-3′; **K270A/E271A/R272A** forward 5′-GAC ATC CAT TCT ACT CGC TCC GCA GCG GCG CTC AAT GCG TAC ACA-3′, reverse 5′-TGT GTA CGC ATT GAG CGC CGC TGC GGA GCG AGT AGA ATG GAT GTC-3′. After the PCR amplification, the *DpnI* endonuclease (New England Biolabs) was used to digest the parental DNA template and to select for the mutation containing newly synthesized DNA. The identity of all mutant constructs was verified by DNA sequencing (Macrogen).

### Hippocampal Cultures, Cell Line, and Plasmid Transfections

Dissociated neuronal cultures were prepared from hippocampi of embryonic day 19 Sprague–Dawley rats obtained from the Faculty of Veterinary Sciences (Buenos Aires, Argentina), as described previously (Alvarez Julia et al., 2016). Briefly, hippocampal tissue was treated with 0.25% (wt/vol) trypsin in HBSGK buffer (4.8 g/l HEPES, 8.7 g/l NaCl, 0.22 g/l KCl, 0.36 g/l glucose, pH 7.4) for 15 min at 37°C. A single-cell solution was prepared in Neurobasal^TM^ medium (Thermo Fisher Scientific) containing 3.5 g/l glucose, 2 mM glutamine, 100 U/ml penicillin, 100 μg/ml streptomycin and 10% (vol/vol) horse serum. Cells were seeded in 24-well plates on coverslips coated with 0.8 mg/ml poly-L-lysine hydrobromide (Sigma-Aldrich) and 5 μg/ml laminin (Thermo Fisher Scientific) at a density of 32,000–37,000 cells/cm^2^. After 2 h at 37°C, medium was changed to a serum-free medium [Neurobasal^TM^ medium supplemented with 3.5 g/l glucose, 2 mM glutamine, 1 g/l ovalbumin, 100 U/ml penicillin, 100 μg/ml streptomycin, N-2 and B-27^TM^ serum-free supplements (Thermo Fisher Scientific)]. All animal procedures were carried out according to the guidelines of NIH Publications No. 80-23 and approved by the Committee for Care and Use of Laboratory Animals, National University of San Martin (CICUAE-UNSAM No. 05/2015, Buenos Aires, Argentina).

Mouse neuroblastoma N2a cells were cultured in Dulbecco’s modified Eagle medium (DMEM) with 20% (vol/vol) fetal bovine serum, 100 U/ml penicillin, 100 μg/ml streptomycin, and 50 μg/ml gentamycin. Cells were seeded on coverslips in 24-well plates at a density of 37,000 cells/cm^2^. For the Western blot and flow cytometry assays, N2a cells were seeded in 6-well plates or 35 mm cell culture dishes at a density of 60,000 cells/cm^2^.

Neuronal cultures or N2a cells in 24-well plates were transiently transfected with 3 μg of DNA mixed with 1 μl of Lipofectamine^®^2000 (Thermo Fisher Scientific) following the manufacturer’s instructions or with 3 μl of polyethylenimine (PEI; Faculty of Pharmacy and Biochemistry, University of Buenos Aires), respectively. N2a cultured in 6-well plates or 35 mm cell culture dishes were transfected with 10 μg of DNA mixed with 15 μl of PEI.

### SDS-Page and Western Blotting

N2a cells transfected in 6-well plates or 35 mm cell culture dishes were rinsed with ice-cold phosphate-buffered saline (PBS) and lysed on ice with lysis buffer [150 mM NaCl, 50 mM Tris-HCl, 1% (wt/vol) deoxycholate sodium salt, 0.1% (wt/vol) SDS, pH 8] supplemented with protease inhibitor cocktail (Sigma-Aldrich) for 15 min. Proteins were precipitated by trichloroacetic acid/acetone precipitation at -20°C overnight. Then, samples were centrifuged at 15,000 *g* for 15 min at 4°C, washed with acetone and centrifuged at 15,000 *g* for 15 min at 4°C. The precipitated proteins were dissolved in rehydration buffer (10 mM dithiothreitol, 20 mM Tris-HCl, pH 6.8, 9 M urea) and the concentration of solubilized proteins was measured using a Bradford assay (Bio-Rad). Finally, 5X SDS sample buffer with 100 mM dithiothreitol was added to each sample. The protein samples were loaded and separated by 10% SDS-polyacrylamide gels (50 μg of total protein/lane) and then transferred to a nitrocellulose membrane by electroblotting. After 1 h of blocking in TBS containing 0.2% (vol/vol) Tween-20 and 2% (vol/vol) fish skin gelatin (FSG), the membranes were incubated with the rabbit anti-GFP polyclonal primary antibody (1/1000, Thermo Fisher Scientific) and the mouse anti-alpha-tubulin monoclonal primary antibody (1/2000, Sigma) overnight. Antigen-antibody complexes were detected by the goat anti-rabbit secondary IRDye800 CW (1/15,000) or the goat anti-mouse secondary IRDye680 LT (1/20,000) (LiCor Biosciences) using Odyssey clx infrared imaging.

### Immunocytochemistry

Cells were fixed in 4% (wt/vol) paraformaldehyde, 4% (wt/vol) sucrose in PBS for 20 min at room temperature. If not indicated otherwise, fixation was followed by permeabilization with 0.1% (vol/vol) Triton X-100 (TX100) in PBS (2 min). Fixed cells were blocked with 3% (wt/vol) bovine serum albumin (BSA) with 0.2% (vol/vol) FSG in PBS and then incubated with primary antibodies in PBS with 1% (wt/vol) BSA (overnight, 4°C) and secondary antibodies (1 h, room temperature). F-actin was stained with rhodamine red-conjugated phalloidin (1/1000; Thermo Fisher Scientific) and nuclei with 4′, 6-diamidino-2-phenylindole dihydrochloride (DAPI) (1/3000) for 5 min at room temperature. Coverslips were mounted in Fluor Save Reagent (Calbiochem).

### Image Acquisition, Analysis, and Quantification

Cells were visualized and fluorescent images were acquired using a Nikon Eclipse 80i [Plan APO 60X oil, 1.4 NA, 0.13 mm working distance (WD) objective] with CoolLED pE excitation system, Nikon E600 microscope with epifluorescence illumination (Plan APO 100X oil, 1.4 NA objective) or Olympus FluoviewFV1000 confocal laser scanning microscope (Plan APO N 60X oil, 1.42 NA, FN 26.5 objective) with FV10-ASW software.

To quantify filopodium formation in N2a cells, F-actin marker phalloidin conjugated with rhodamine red was used to visualize filopodia and the percentage of transfected cells showing filopodia was calculated. Each experiment was scored blind. On average, 90–120 cells for each transfection condition done in duplicates were analyzed in randomly selected regions in multiple independent experiments.

In primary hippocampal neurons, filopodium density (the number of filopodia per 45 μm neurite length) was quantified as described previously (Alvarez Julia et al., 2016) in 35–55 neurites of 10–20 different neurons from each transfection condition done in duplicates in at least two independent experiments. Each experiment was scored blind. Previously we have shown that all filopodial protrusions induced by Gpm6a overexpression are labeled by phalloidin (Alvarez Julia et al., 2016) and that quantification of phalloidin-labeled filopodia coincided with quantification when EGFP is used to detect filopodia in transfected neurons (Alvarez Julia et al., 2016).

Colocalization was analyzed using Colocalization Analysis plugins (ImageJ software). First, the Colocalization Test plugin with Fay randomization method was performed to calculate Pearson’s correlation coefficient for the two channels in each selected ROI (25 × 25 pixels). This value was compared with what would be expected for random overlap. The observed correlation was considered significant if it was greater than 95% of the correlations between channel 1 and a number of randomized channel 2 images. All ROIs with *p* value for Pearson’s coefficient ≥ 0.95 were further analyzed by the Colocalization Threshold plugin to calculate thresholded Mander’s coefficients [tM1 colocalization value for channel 1 (red); tM2 colocalization value for channel 2 (green)] and to generate scatterplots with linear regression line and thresholds. On average, 2–3 regions of interest from each cell in 10–15 neurons from each transfection condition done in duplicates were analyzed in two independent experiments. Each experiment was scored blind.

### Flow Cytometry

N2a cells cultured in 6-well plates or 35 mm cell culture dishes were gently harvested by scraping and incubated on ice with the rat anti-Gpm6a (MBL, 1/250) in 100 μl of ice-cold PBS for 1 h, followed by the incubation with the goat anti-rat IgG conjugated to AF 647 (1/500, BioLegend) in the final volume of 100 μl of ice-cold PBS. After 1 h, cells were washed, fixed with 2% paraformaldehyde in PBS for 20 min, and analyzed by flow cytometry. Non-transfected cells incubated only with the secondary antibody were included as a control. All procedures were done at 4°C. Initial control experiments using the immunofluorescence microscopy were performed to confirm that the antibody does not cross the plasma membrane and only the surface exposed antigen is labeled using the procedure described above (**Supplementary Figure S3**).

FlowMax cytometer Particle Analyzing System PAS-III (Sysmex Partec GmbH, Gorlitz, Germany) and FlowJo software (FlowJo V10, Ashland, OR, United States) were used throughout this work for acquisition of events and data analysis. The gating of N2a cells population was done using a forward scatter (FCS) and side scatter (SSC) plot. This population was further evaluated for total EGFP expression and Gpm6a surface expression (**Supplementary Figure S2**).

### Statistical Analysis

Group means were analyzed for overall statistical significance using one-way ANOVA followed by Tukey’s or Dunnett’s multiple comparison tests for *post hoc* effects. Results are reported as means ± SEM. For all tests, *p* ≤ 0.05 was considered statistically significant. Calculations and graphs were done with GraphPad Prism 6.00.

## Results

### The C-Terminal but Not the N-Terminal Cytosolic End of Gpm6a Is Required for the Process of Gpm6a-Induced Filopodium Formation

**FIGURE 1:**
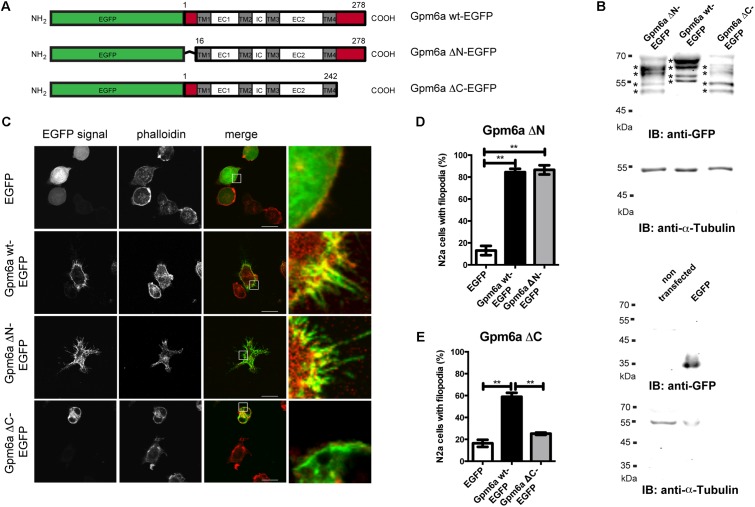
Deletion of the C-terminal but not the N-terminal intracellular domain of Gpm6a interferes with filopodium formation in neuroblastoma cell line N2a. **(A)** Schematic structure of the EGFP-tagged wild type (wt) Gpm6a and Gpm6a deletion constructs: Gpm6a wt-EGFP (full length wt Gpm6a, 1–278 aa), Gpm6a ΔN-EGFP (Δ1–16), Gpm6a ΔC-EGFP (Δ243–278). The domain organization is indicated by colored boxes: the N-terminal and the C-terminal intracellular domains (red); the four transmembrane domains (TM1-4; gray); the intracellular (IC), the small extracellular (EC1), and the large extracellular (EC2) loops (white). **(B)** Western blot of lysates from N2a cells overexpressing the indicated constructs. Immunnoblot (IB) was analyzed using the rabbit anti-GFP antibody detected by the goat anti-rabbit secondary IRDye800 CW. Bands representing Gpm6a proteins are indicated by asteriscs. As a loading control alpha-tubulin was detected using the mouse anti-alpha-tubulin monoclonal antibody followed by the goat anti-mouse secondary IRDye680 LT. Below, the control Western blot of lysates from non-transfected N2a cells or N2a overexpressing EGFP alone shows the specificty of detected signal. **(C)** Localization of ΔN and ΔC Gpm6a mutants in N2a cells. Confocal images of N2a cells transfected with the indicated vectors and labeled with rhodamine red-conjugated phalloidin to visualize F-actin cytoskeleton. Gpm6a wt-EGFP and EGFP alone were used as controls. Gpm6a wt-EGFP and Gpm6a ΔN-EGFP accumulate at plasma membrane and in filopodial protrusions (second and third row). Gpm6a ΔN-EGFP and Gpm6a ΔC-EGFP show higher cytoplasmic localization comparing to the wt Gpm6a. Overexpression of Gpm6a ΔC-EGFP does not induce filopodia formation (bottom row). Scale bar, 10 μm. **(D,E)** The percentage of transfected N2a cells showing filopodia was quantified in red channel visualizing rhodamine red-phalloidin. On average, 97–119 cells for each transfection condition done in duplicates were analyzed in multiple experiments. Data are means ± SEM. One-way ANOVA followed by Tukey’s multiple comparison test for *post hoc* effects. **(D)** Gpm6a ΔN-EGFP: ^∗∗^*p* < 0.01 EGFP vs Gpm6a wt-EGFP, ^∗∗^*p* < 0.01 EGFP vs Gpm6a ΔN-EGFP. **(E)** Gpm6a ΔC-EGFP: ^∗∗^*p* < 0.01 EGFP vs Gpm6a wt-EGFP, ^∗∗^*p* < 0.01 Gpm6a wt-EGFP vs Gpm6a ΔC-EGFP. No statistically significant differences between EGFP and Gpm6a ΔC-EGFP were detected.

Filopodium formation is one of the processes driven by Gpm6a. To map the domains of the Gpm6a molecule that are required for this process, we generated Gpm6a deletion mutants lacking the N-terminal (ΔN, amino acids 1–16) or the C-terminal (ΔC, amino acids 243–278) intracellular domains as shown in the **Figure 1A**. To determine whether deletion of these sequences interferes with the expression of the proteins, lysates from neuroblastoma cell line N2a overexpressing the ΔN, the ΔC deletion mutants, and the wt Gpm6a tagged with EGFP were analyzed on immunoblots using anti-GFP antibody (**Figure 1B**). The Western blot in the **Figure 1B** shows that as reported previously (Fuchsova et al., 2009; Alvarez Julia et al., 2016), the exogenous wt Gpm6a fused to EGFP migrates as multiple bands at 62–67 kDa most probably due to posttranslational modifications of the protein such as phosphorylation and glycosylation (**Figure 1B**, lane 2). The same pattern of multiple bands migrating at lower size is observed for both the Gpm6a ΔN-EGFP and the Gpm6a ΔC-EGFP deletion mutants (**Figure 1B**, lanes 1 and 3, respectively). This indicates that the deletion mutants are successfully expressed in N2a cells and their migration on SDS-PAGE is consistent with the lower size of the truncated mutant proteins.

Next, we examined the subcellular localization of the truncated mutant proteins and the effect of their overexpression on cell morphology. Overexpression experiments were first performed in N2a cells labeled with F-actin marker phalloidin to visualize filopodia. Overexpression of Gpm6a wt-EGFP and EGFP alone were used as controls. **Figure 1C** demonstrates that upon overexpression, Gpm6a wt-EGFP and Gpm6a ΔN-EGFP accumulate at the plasma membrane and in filopodial protrusions. When compared to the wt Gpm6a, both the Gpm6a ΔN-EGFP and the Gpm6a ΔC-EGFP show higher accumulation in the cytosol (**Figure 1C**). As described in our previous work (Alfonso et al., 2005; Alvarez Julia et al., 2016), overexpression of Gpm6a wt-EGFP leads to a significant increase in the formation of filopodia comparing to the control expression of EGFP alone (**Figure 1C**, first and second row). Filopodium formation is also observed upon overexpression of the N-terminal Gpm6a deletion mutant lacking amino acids 1–16; Gpm6a ΔN-EGFP (**Figure 1C**, third row). In contrast, Gpm6a lacking C-terminal intracellular domain (amino acids 243–278; Gpm6a ΔC-EGFP) does not induce filopodium formation (**Figure 1C**, bottom row). Quantification of the percentage of cells showing filopodia revealed that upon Gpm6a ΔC-EGFP overexpression, filopodium formation was significantly lower when compared to the wt Gpm6a (**Figure 1E**). On the other hand, Gpm6a ΔN-EGFP induced filopodium formation to the same extent as the wt Gpm6a (**Figure 1D**).

**FIGURE 2:**
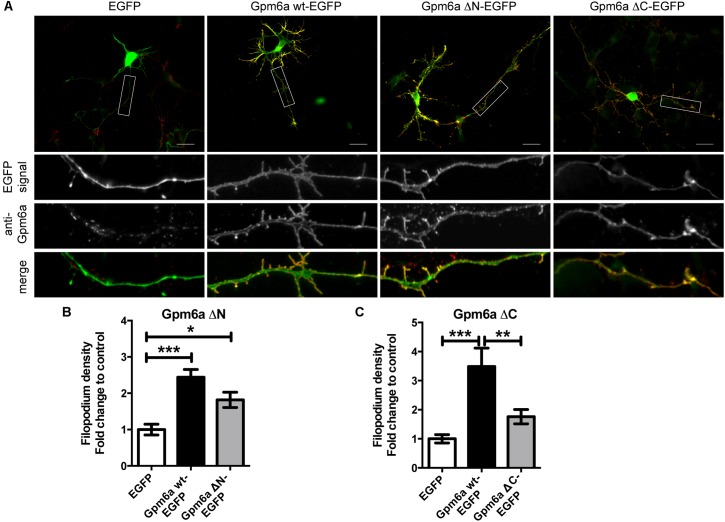
Deletion of the C-terminal intracellular domain of Gpm6a interferes with filopodium formation in hippocampal neurons but a function blocking anti-Gpm6a antibody recognizes surface exposed epitopes of both the ΔN and the ΔC Gpm6a. **(A)** Micrographs of primary hippocampal neurons (4 DIV) transfected with the indicated vectors and immunostained with rat anti-Gpm6a mAb in non-permeabilized cells. Goat anti-rat IgG labeled with rhodamine red was used as a secondary antibody. Maximized views of neurites show that the surface-exposed regions of Gpm6a wt-EGFP as well as both the Gpm6a ΔN-EGFP and the Gpm6a ΔC-EGFP are recognized by the anti-Gpm6a antibody. Scale bar, 20 μm. **(B,C)** Filopodium density (the number of protrusions per 45-μm of neurite length) as shown in the maximized views was quantified. Data are means ± SEM. Twenty to thirty three neurons per group done in duplicates were analyzed in multiple independent experiments. One-way ANOVA followed by Tukey’s multiple comparison test for *post hoc* effects, **(B)** Gpm6a ΔN-EGFP: ^∗∗∗^*p* < 0.001 EGFP vs Gpm6a wt-EGFP, ^∗^*p* < 0.05 EGFP vs Gpm6a ΔN-EGFP. **(C)** Gpm6a ΔC-EGFP: ^∗∗∗^*p* < 0.001 EGFP vs Gpm6a wt-EGFP, ^∗∗^p < 0.01 Gpm6a wt-EGFP vs Gpm6a ΔC-EGFP. No statistically significant differences between EGFP and Gpm6a ΔC-EGFP were detected.

Next, we evaluated the effect of the overexpression of the truncated forms of Gpm6a on filopodium formation in primary hippocampal neurons. Neurons of 3 DIV were transfected with indicated constructs (**Figure 2A**). Overexpression of Gpm6a wt-EGFP and EGFP alone were used as controls. **Figure 2A** demonstrates that the overexpression of wt Gpm6a leads to a significant increase in filopodium density when compared to the control overexpression of EGFP alone. As observed for N2a cells, transfection with Gpm6a ΔN-EGFP also resulted in an increase in filopodium density (**Figure 2A**). On the other hand, overexpression of the mutant Gpm6a ΔC-EGFP did not lead to the induction of filopodium formation (**Figure 2A**). Filopodium density (number of protrusions per 45-μm of neurite length) as shown in the enlarged pictures (**Figure 2A**) was quantified. Neurons overexpressing Gpm6a ΔN-EGFP have filopodium density significantly higher than control neurons overexpressing EGFP alone (**Figure 2B**). When compared to the wt Gpm6a, the induction of filopodia is lower reaching approximately 75% of the effect induced by wt Gpm6a but the difference does not reach statistical significance (**Figure 2B**). On the other hand, filopodium density of neurons overexpressing Gpm6a ΔC-EGFP is significantly lower (approximately 50%) comparing to neurons expressing the wt Gpm6a and does not significantly differ from the control EGFP (**Figure 2C**). Thus, it can be concluded that the deletion of the C-terminal, but not the N-terminal, cytosolic end of Gpm6a interferes with Gpm6a-induced filopodium outgrowth in N2a cells as well as in primary hippocampal neurons.

### Deletion of the N- or the C-Terminal Intracellular Domains Does Not Prevent the Recognition of Gpm6a by a Function Blocking Anti-Gpm6a Antibody but Diminishes the Amount of Gpm6a on Cell Surface

Previous studies reported that the monoclonal anti-Gpm6a antibody directed against the large extracellular loop (EC2) of Gpm6a affects function of the protein (Lagenaur et al., 1992; Sato et al., 2011; Formoso et al., 2015). We asked whether deletion of the N- or C-terminal intracellular domains of Gpm6a would interfere with binding of this monoclonal antibody. **Figure 2A** shows images of the non-permeabilized primary hippocampal neurons of 4 DIV transfected with the indicated EGFP-tagged mutants and immunostained with the rat anti-Gpm6a mAb (**Figure 2A**). We observed that the surface-exposed epitopes of EGFP-tagged wt Gpm6a as well as both the Gpm6a ΔN-EGFP and the Gpm6a ΔC-EGFP are recognized by the anti-Gpm6a antibody (**Figure 2A**, maximized views of neurites). The colocalization can be observed in the merge images. We conclude that the deletion of the N- or the C-terminal intracellular domains does not prevent the localization of Gpm6a on cell surface nor it modifies conformation of the large extracellular loop so it can be recognized by the function blocking anti-Gpm6a antibody.

**FIGURE 3:**
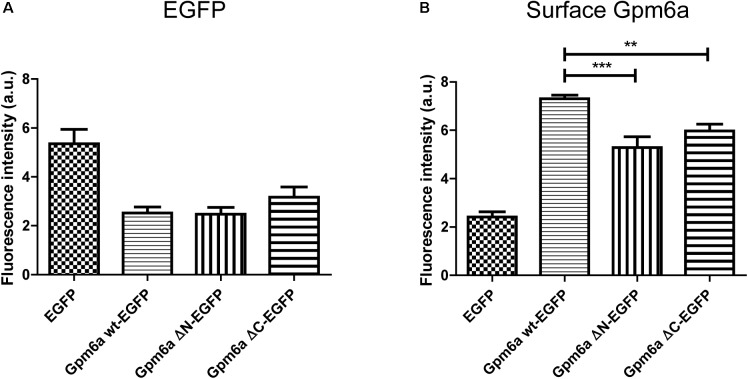
Deletion of the N- or the C-terminal intracellular domain diminishes the amount of Gpm6a on cell surface. **(A)** Flow cytometry was used to measure and calculate the mean fluorescence intensity of EGFP in the population of EGFP-positive N2a cells transfected with the indicated vectors as a measure of the total amount of EGFP-tagged proteins. Data are means ± SEM of two independent experiments. One-way ANOVA followed by Dunnett’s multiple comparisons test for *post hoc* effects revealed no significant differences between Gpm6a wt-EGFP vs Gpm6a ΔN-EGFP and Gpm6a wt-EGFP vs Gpm6a ΔC-EGFP. **(B)** The mean fluorescence intensity of the surface-labeled Gpm6a measured by flow cytometry in the population of EGFP-positive N2a cells transfected with the indicated vectors. Surface Gpm6a was labeleld by immunostaining of non-permeabilized cells with the rat anti-Gpm6a antibody followed by goat anti-rat IgG conjugated to Alexa Fluor 647. Data are means ± SEM of two independent experiments. One-way ANOVA followed by Dunnett’s multiple comparisons test for *post hoc* effects, ^∗∗∗^*p* < 0.001 Gpm6a wt-EGFP vs Gpm6a ΔN-EGFP and ^∗∗^*p* < 0.01 Gpm6a wt-EGFP vs Gpm6a ΔC-EGFP.

Next we asked whether deletion of the N- or the C- terminal sequences affects the expression levels of mutant proteins or their amount on cell surface. To quantify these parameters we have employed flow cytometry in N2a cells transfected with the indicated EGFP-tagged mutants. EGFP-tagged wt Gpm6a, EGFP alone and non-transfected cells were used as controls. Twenty four hours after transfection, the cells were fixed at 4°C to prevent membrane trafficking and Gpm6a present on cell surface was labeled by immunostaining of non-permeabilized cells with the rat anti-Gpm6a mAb. Goat anti-rat IgG conjugated to Alexa Fluor 647 was used as a secondary antibody. The fluorescence intensity of the non-transfected intact cells immunostained only with the secondary antibody was used as a control to define EGFP and Gpm6a positive N2a populations and their percentage (**Supplementary Figure S2**). The number of the EGFP-positive and the EGFP-negative cells for each condition was counted. For Gpm6a wt-EGFP ∼13% (SEM ± 0.623) of the total cells assessed was EGFP-positive, for Gpm6a ΔN-EGFP ∼17% (SEM ± 0.8111), and for Gpm6a ΔC-EGFP ∼17% (SEM ± 1.103) suggesting that the transfection efficiency was not negatively affected by overexpression of vectors bearing deletions. The intensity of the fluorescence signal of EGFP as a measure of the amount of expressed protein was quantified in the fraction of EGFP-positive cells (**Figure 3A**). No significant differences were observed for Gpm6a ΔN-EGFP nor for Gpm6a ΔC-EGFP when compared to Gpm6a wt-EGFP (**Figure 3A**). When the intensity of the fluorescence signal of the surface Gpm6a was quantified in the fraction of EGFP-positive cells, lower amount of Gpm6a present on the cell surface was observed for both the Gpm6a ΔN-EGFP and the Gpm6a ΔC-EGFP displaying 27.7% and 18.4% less surface Gpm6a, respectively, comparing to the wt Gpm6a (**Figure 3B**). These results indicate that deletion of both terminal intracellular domains of Gpm6a leads to the lower amount of Gpm6a protein on cell surface. We suppose that proper protein folding and/or cell surface trafficking could be partially affected by these deletions. Nevertheless, no differences were detected between the Gpm6a ΔN-EGFP and the Gpm6a ΔC-EGFP suggesting that the incapacity of the Gpm6a ΔC-EGFP to induce filopodium formation is not merely due to the lower amount of Gpm6a present on cell surface.

### Alanine Scanning Mutagenesis of the C-Terminal Cytosolic End of Gpm6a Reveals Key Residues for the Process of Filopodia Formation

**FIGURE 4:**
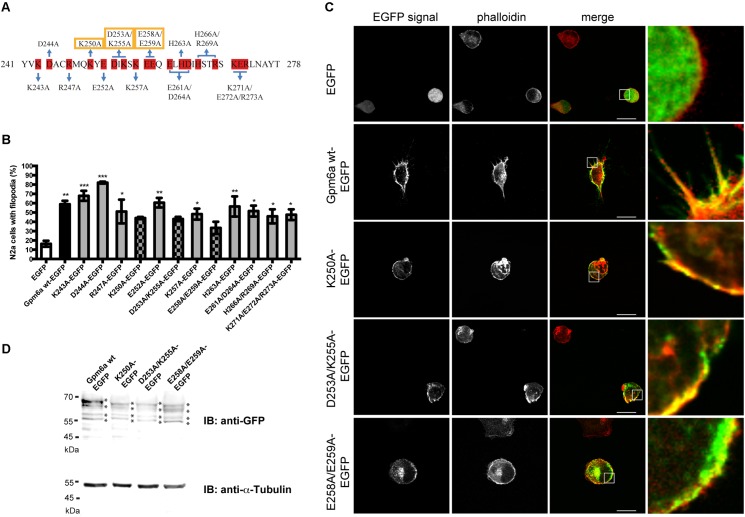
Alanine scanning mutagenesis of the C-terminal cytosolic end of Gpm6a. **(A)** Amino acid sequence of the C-terminal end (aa 241–278) of the mouse Gpm6a (NCBI Accession: NP_705809.1). Charged amino acids substituted with alanine are highlighted in red. Blue lines indicate charged residues mutated simultaneously. Residues that affect filopodium formation when mutated to alanine are indicated by yellow boxes. **(B)** N2a cells transfected with the indicated vectors and labeled with rhodamine red-conjugated phalloidin to visualize F-actin cytoskeleton were analyzed by fluorescence microscopy. The percentage of transfected N2a cells showing filopodia was quantified in red channel visualizing rhodamine red-phalloidin. On average, 97–125 cells for each transfection condition done in duplicates were analyzed. Data are means ± SEM. One-way ANOVA followed by Dunnett’s multiple comparison test for *post hoc* effects, ^∗^*p* < 0.05 EGFP vs R247A-EGFP, ^∗^*p* < 0.05 EGFP vs K257A-EGFP, ^∗^*p* < 0.05 EGFP vs E261A/D264A-EGFP, ^∗^*p* < 0.05 EGFP vs H266A/R269A-EGFP, ^∗^*p* < 0.05 EGFP vs K271A/E272A/R273A-EGFP, ^∗∗^*p* < 0.01 EGFP vs Gpm6a wt-EGFP, ^∗∗^*p* < 0.01 EGFP vs E252A-EGFP, ^∗∗^*p* < 0.01 EGFP vs H263A-EGFP, ^∗∗∗^*p* < 0.001 EGFP vs K243A-EGFP, ^∗∗∗^*p* < 0.001 EGFP vs D244A-EGFP. One-way ANOVA revealed no significant differences for EGFP vs K250-EGFP, EGFP vs D253A/K255A-EGFP, and EGFP vs E258A/E259A-EGFP. **(C)** Localization of K250-EGFP, D253A/K255A-EGFP, and E258A/E259A-EGFP mutants in N2a cells. Confocal images of N2a cells transfected with the indicated vectors and labeled with rhodamine red-conjugated phalloidin. Gpm6a wt-EGFP and EGFP alone were used as controls. K250A, D253A/K255A, and E258A/E259A localize to the plasma membrane of N2a but fail to form filopodial protrusions. Scale bar, 10 μm. **(D)** Western blot of lysates from N2a cells overexpressing the indicated constructs. Immunnoblot (IB) was analyzed using the rabbit anti-GFP antibody detected by the goat anti-rabbit secondary IRDye800 CW. Bands representing Gpm6a proteins are indicated by asteriscs. As a loading control alpha-tubulin was detected using the mouse anti-alpha-tubulin monoclonal antibody followed by the goat anti-mouse secondary IRDye680 LT.

To identify functionally critical residues within the C-terminus of Gpm6a, a charged-to-alanine scanning mutagenesis (Gibbs and Zoller, 1991) was used to construct a panel of 12 Gpm6a mutants where all charged amino acids in the C-terminal cytosolic end were systematically substituted with alanine as described in Methods section (**Figure 4A**).

As a primary screen for functional residues, a collection of generated EGFP-tagged mutant proteins was evaluated for the effect of their overexpression on filopodium formation in N2a cells. Labeling with F-actin marker phalloidin conjugated with rhodamine red was employed to visualize filopodia. Overexpression of Gpm6a wt-EGFP and EGFP alone were used as controls. **Figure 4B** shows the quantification of the percentage of transfected N2a cells showing filopodia. We observed that only three out of 12 mutants (K250A, D253A/K255A, and E258A/E259A) do not induce formation of filopodia significantly higher than control EGFP (**Figure 4B**). Confocal micrographs in the **Figure 4C** show that upon their overexpression, these three mutants (K250A, D253A/K255A, and E258A/E259A) localize to the plasma membrane but fail to form filopodial protrusions (**Figure 4C**). When cell lysates of N2a cells transfected with K250A, D253A/K255A, and E258A/E259A were analyzed on Western blot using anti-GFP antibody, the same pattern of multiple bands migrating at the same size as Gpm6a wt-EGFP was observed for all three mutants (**Figure 4D**) indicating that K250A, D253A/K255A, and E258A/E259A mutant proteins are successfully expressed in N2a cells and their migration on SDS-PAGE is consistent with the size expected.

**FIGURE 5:**
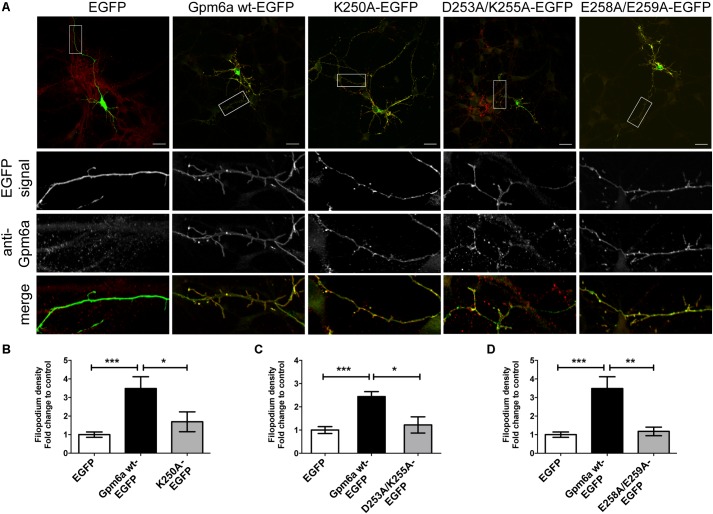
Substitution with alanine of K250, D253/K255, and E258/E259 of Gpm6a interferes with filopodium formation in primary hippocampal neurons but does not prevent mutant recognition by the function blocking anti-Gpm6a antibody. **(A)** Micrographs of primary hippocampal neurons (4 DIV) transfected with the indicated vectors and immunostained with rat anti-Gpm6a mAb in non-permeabilized cells. Goat anti-rat IgG labeled with rhodamine red was used as a secondary antibody. Maximized views of neurites show that the surface-exposed epitopes of the wt Gpm6a and the mutant proteins K250A, D253A/K255A, and E258A/E259A are recognized by the anti-Gpm6a antibody. Scale bar, 20 μm. **(B–D)** Filopodium density (the number of protrusions per 45-μm of neurite length) as shown in the maximized views was quantified. Data are means ± SEM. Ten to twenty neurons per group done in duplicates were analyzed in two independent experiments. One-way ANOVA followed by Tukey’s multiple comparison test for *post hoc* effects. **(B)** K250A-EGFP: ^∗∗∗^*p* < 0.001 EGFP vs Gpm6a wt-EGFP, ^∗^*p* < 0.05 Gpm6a wt-EGFP vs K250A-EGFP. **(C)** D253A/K255A-EGFP: ^∗∗∗^*p* < 0.001 EGFP vs Gpm6a wt-EGFP, ^∗^*p* < 0.05 Gpm6a wt-EGFP vs D253A/K255A. **(D)** E258A/E259A-EGFP: ^∗∗∗^*p* < 0.001 EGFP vs Gpm6a wt-EGFP, ^∗∗^*p* < 0.01 Gpm6a wt-EGFP vs E258A/E259A-EGFP. No statistically significant differences between EGFP and K250A-EGFP **(B)**, EGFP and D253A/K255A-EGFP **(C)**, EGFP and E258A/E259A-EGFP **(D)** were detected.

Three mutants displaying deficiencies in the formation of filopodia were further evaluated in primary hippocampal neurons. Neurons of 3 DIV were transfected with indicated constructs and fixed 24 h later (**Figure 5A**). Overexpression of Gpm6a wt-EGFP and EGFP alone were used as controls. **Figure 5A** shows that the overexpression of Gpm6a wt-EGFP significantly increased filopodium density comparing to the control overexpression of EGFP alone. On the other hand, neurons expressing K250A, D253A/K255A, or E258A/E259A mutants displayed decreased filopodium number (**Figure 5A**). The quantification results in the **Figures 5B–D** show that the filopodium density of neurons overexpressing K250A, D253A/K255A, and E258A/E259A mutants does not significantly differ from the control EGFP and is significantly lower comparing to neurons expressing the wt Gpm6a. Thus, it can be concluded that the substitution with alanine of charged amino acids K250, D253/K255, and E258/E259 in the C-terminal cytosolic end of Gpm6a interferes with Gpm6a-induced filopodium outgrowth in N2a cells as well as in primary hippocampal neurons.

### Gpm6a Mutant Proteins K250A, D253A/K255A, and E258A/E259A Localize at Cell Surface and Are Recognized by a Function Blocking Anti-Gpm6a Antibody

Next, we evaluated whether the substitution of charged amino acids K250, D253/K255, and E258/E259 with alanine would interfere with binding of the anti-Gpm6a antibody that recognizes the surface-exposed epitope. Primary hippocampal neurons of 3 DIV were transfected with the indicated EGFP-tagged mutants. Immunostaining was performed in non-permeabilized cells with rat anti-Gpm6a mAb. EGFP tagged wt Gpm6a and EGFP alone were used as controls. We show that the surface-exposed regions of EGFP-tagged wt Gpm6a as well as all three mutant proteins K250A, D253A/K255A, and E258A/E259A are recognized by the anti-Gpm6a antibody in hippocampal neurons (**Figure 5A**, maximized views of neurites). The colocalization can be observed in the merge images. Thus, mutation to alanine of K250, D253/K255, and E258/E259 of Gpm6a does not prevent the localization of Gpm6a on cell surface nor it modifies conformation of the large extracellular loop so it can be recognized by a function blocking anti-Gpm6a antibody.

**FIGURE 6:**
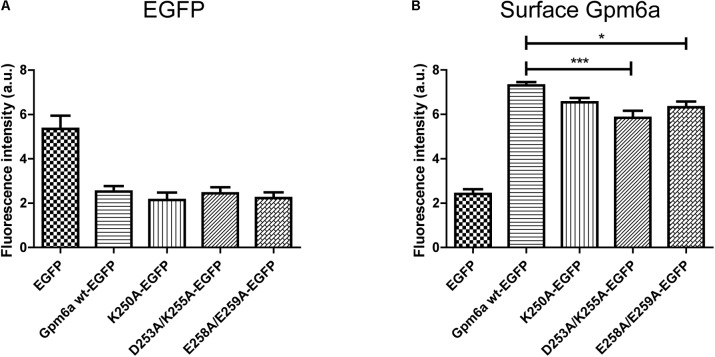
Substitution with alanine of K250, D253/K255, and E258/E259 diminishes the amount of Gpm6a on cell surface. **(A)** Flow cytometry was used to measure and calculate the mean fluorescence intensity of EGFP in the population of EGFP-positive N2a cells transfected with the indicated vectors as a measure of the total amount of EGFP-tagged proteins. Data are means ± SEM of three independent experiments. One-way ANOVA followed by Dunnett’s multiple comparisons test for *post hoc* effects revealed no significant differences between Gpm6a wt-EGFP vs K250A-EGFP, Gpm6a wt-EGFP vs D253A/K255A-EGFP, and Gpm6a wt-EGFP vs E258A/E259A-EGFP. **(B)** The mean fluorescence intensity of the surface-labeled Gpm6a measured by flow cytometry in the population of EGFP-positive N2a cells transfected with the indicated vectors. Surface Gpm6a was labeleld by immunostaining of non-permeabilized cells with the rat anti-Gpm6a antibody followed by goat anti-rat IgG conjugated to Alexa Fluor 647. Data are means ± SEM of three independent experiments. One-way ANOVA followed by Dunnett’s multiple comparisons test for *post hoc* effects, ^∗∗∗^*p* < 0.001 Gpm6a wt-EGFP vs D253A/K255A-EGFP, ^∗^*p* < 0.05 Gpm6a wt-EGFP vs E258A/E259A-EGFP, *p* = 0.0544 Gpm6a wt-EGFP vs K250A-EGFP.

The protein expression levels and the amount of Gpm6a present on cell surface were then quantified by flow cytometry in N2a cells transfected with the indicated EGFP-tagged mutants as described in the previous section. For K250A-EGFP ∼14% (SEM ± 2.207) of the total cells assessed was EGFP-positive, for D253A/K255A-EGFP ∼15% (SEM ± 1.38), and for E258A/E259A-EGFP ∼13% (SEM ± 1.99), indicating that the transfection efficiency of mutants did not differ from that of Gpm6a wt-EGFP (∼13%, SEM ± 0.623). The intensity of the fluorescence signal of EGFP as a measure of the amount of expressed protein was quantified in the fraction of EGFP-positive cells (**Figure 6A**). No significant differences were observed for mutant proteins K250A, D253A/K255A, and E258A/E259A when compared to Gpm6a wt-EGFP (**Figure 6A**). When the intensity of the fluorescence signal of the surface Gpm6a was quantified in the fraction of EGFP-positive cells, mutant proteins K250A, D253A/K255A, and E258A/E259A displayed 10.5, 20.1, and 13.5% less Gpm6a present on the cell surface, respectively, when compared to the wt Gpm6a (**Figure 6B**). The difference was statistically significant for D253A/K255A and E258A/E259A and showed tendency toward significance for K250A (*p* = 0.0544). These results indicate that the substitutions of charged amino acids K250, D253/K255, and E258/E259 with alanine interfere to a certain extent with the amount of the Gpm6a protein on cell surface, probably due to the destabilizing effect on protein folding and/or cell surface trafficking of the protein.

### Functional Effects of Separate Substitutions With Alanine of D253, K255, E258, and E259 in the Gpm6a C-Terminal Cytosolic End

To dissect the functional information on residues that when mutated in conjunction (D253A/K255A and E258A/E259A) displayed functional defects, we next constructed Gpm6a mutants with separate substitution of D253, K255, E258, and E259 with alanine.

**FIGURE 7:**
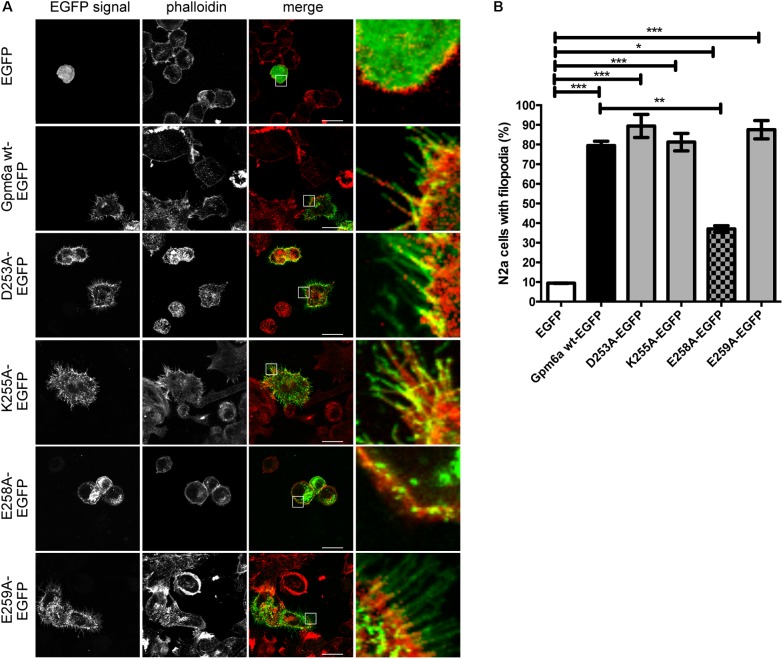
Effect on filopodium formation of separate substitution with alanine of D253, K255, E258, and E259 in the C-terminal cytosolic end of Gpm6a in neuroblastoma cell line N2a. **(A)** Confocal images of N2a cells transfected with the indicated vectors and labeled with rhodamine red-conjugated phalloidin to visualize F-actin cytoskeleton. Gpm6a wt-EGFP and EGFP alone were used as controls. D253A, K255A, and E259A localize to the plasma membrane of N2a and induce formation of filopodia similarly to the wt Gpm6a. The formation of filopodia is reduced upon overexpression of E258A when compared to the wt Gpm6a. Scale bar, 10 μm. **(B)** The percentage of transfected N2a cells showing filopodia was quantified in red channel visualizing rhodamine red-phalloidin. On average, 137–181 cells for each transfection condition done in duplicates were analyzed in multiple experiments. Data are means ± SEM. One-way ANOVA followed by Tukey’s multiple comparison test for *post hoc* effects. ^∗∗∗^*p* < 0.001 EGFP vs Gpm6a wt-EGFP, ^∗∗∗^*p* < 0.001 EGFP vs D253A-EGFP, ^∗∗∗^*p* < 0.001 EGFP vs K255A-EGFP, ^∗∗∗^*p* < 0.001 EGFP vs E259A-EGFP, ^∗^*p* < 0.05 EGFP vs E258A-EGFP, ^∗∗^*p* < 0.01 Gpm6a wt-EGFP vs E258A-EGFP.

The EGFP-tagged mutant proteins D253A, K255A, E258A, and E259A were first evaluated for the effect of their overexpression on filopodium formation in N2a cells. Overexpression of Gpm6a wt-EGFP and EGFP alone were used as controls. Confocal micrographs in the **Figure 7A** show that upon their overexpression, all four mutant proteins localize to the plasma membrane and also display some accumulation in the cytosol, E258A in particular. **Figure 7B** shows the quantification of the percentage of transfected N2a cells showing filopodia. The formation of filopodia is significantly higher for D253A, K255A, and E259A when compared to control EGFP and does not differ from the wt Gpm6a. In contrast, upon E258A overexpression, the percentage of N2a cells with filopodia is significantly lower when compared to the wt Gpm6a (**Figure 7B**).

**FIGURE 8:**
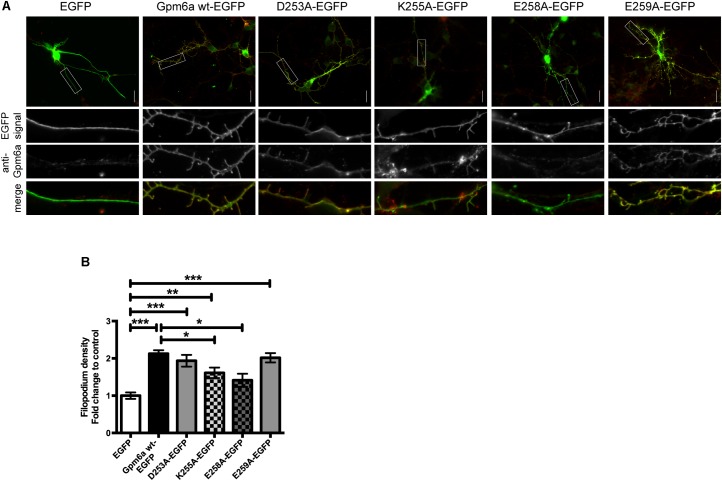
Effect of separate substitution with alanine of D253, K255, E258, and E259 of Gpm6a on filopodium formation in primary hippocampal neurons and recognition of the mutant proteins by the function blocking anti-Gpm6a antibody. **(A)** Micrographs of primary hippocampal neurons (4 DIV) transfected with the indicated vectors and immunostained with rat anti-Gpm6a mAb in non-permeabilized cells. Goat anti-rat IgG labeled with rhodamine red was used as a secondary antibody. Maximized views of neurites show that the surface-exposed epitopes of the wt Gpm6a and the mutant proteins D253A, K255A, and E259A are recognized by the anti-Gpm6a antibody. Only weak labeling of E258A by anti-Gpm6a mAb was detected. Scale bar, 20 μm. **(B)** Filopodium density (the number of protrusions per 45-μm of neurite length) as shown in the maximized views was quantified. Ten to twenty neurons per group done in duplicates were analyzed. Data are means ± SEM of six independent experiments. One-way ANOVA followed by Dunnett’s multiple comparison test for *post hoc* effects. ^∗∗∗^*p* < 0.001 EGFP vs Gpm6a wt-EGFP, ^∗∗∗^*p* < 0.001 EGFP vs D253A-EGFP, ^∗∗^*p* < 0.01 EGFP vs K255A-EGFP, ^∗∗∗^*p* < 0.001 EGFP vs E259A-EGFP, ^∗^*p* < 0.05 Gpm6a wt-EGFP vs K255A-EGFP, ^∗^*p* < 0.05 Gpm6a wt-EGFP vs E258A-EGFP.

The mutants were further evaluated for filopodium formation in primary hippocampal neurons. Images of primary hippocampal neurons of 3 DIV transfected with the indicated EGFP-tagged mutants are shown in the **Figure 8A**. The filopodium density of neurons overexpressing D253A and E259A mutants does not significantly differ from the wt Gpm6a and is significantly higher comparing to neurons expressing control EGFP. On the other hand, K255A and E258A display significantly lower filopodium density comparing to the wt Gpm6a. When compared to the control EGFP, K255A overexpression leads to increased filopodium density while E258A does not show any difference (**Figure 8B**). We conclude that the separate substitution with alanine of charged amino acid E258 in the C-terminal cytosolic end of Gpm6a interferes with Gpm6a-induced filopodium outgrowth in N2a cells as well as in primary hippocampal neurons. On the other hand, upon separate substitution of K255 with alanine, the effect is observed only in hippocampal neurons. No effect is observed on filopodium formation upon substitution of D253 and E259.

### Localization on the Cell Surface and Recognition by the Function Blocking Anti-Gpm6a Antibody of Gpm6a Mutant Proteins D253A, K255A, E258A, and E259A

Next, we evaluated whether the separate substitution of charged amino acids D253, K255, E258, and E259 with alanine would interfere with binding of the anti-Gpm6a antibody that recognizes the surface-exposed epitope. Primary hippocampal neurons of 3 DIV were transfected with the indicated EGFP-tagged mutants. Immunostaining was performed in non-permeabilized cells with rat anti-Gpm6a mAb. EGFP-tagged wt Gpm6a and EGFP alone were used as controls. We show that the surface-exposed regions of the EGFP-tagged wt Gpm6a as well as mutant proteins D253A, K255A, and E259A are recognized by the anti-Gpm6a antibody in non-permeablized hippocampal neurons. The colocalization can be observed in the merge images. On the other hand, a very weak labeling of E258A by anti-Gpm6a mAb was observed (**Figure 8A**, maximized views of neurites). Thus, mutation to alanine of E258, but not D253, K255, and E259, prevents the localization of Gpm6a on cell surface or modifies the conformation of the large extracellular loop.

**FIGURE 9:**
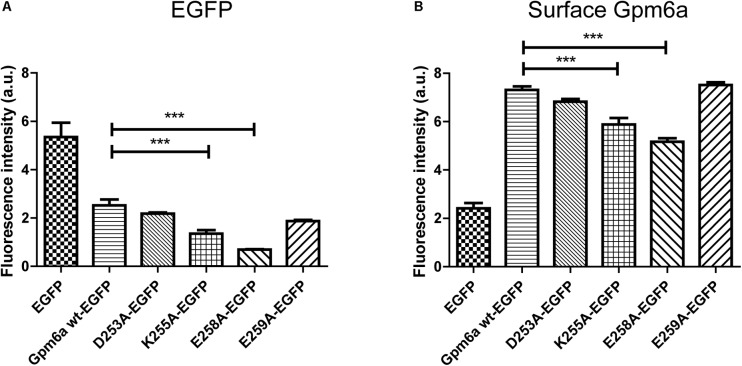
Substitution with alanine of K255 and E258 diminishes the amount of expressed protein and the amount of Gpm6a on cell surface. **(A)** Flow cytometry was used to measure and calculate the mean fluorescence intensity of EGFP in the population of EGFP-positive N2a cells transfected with the indicated vectors as a measure of the total amount of EGFP-tagged proteins. Data are means ± SEM of three independent experiments. One-way ANOVA followed by Dunnett’s multiple comparisons test for *post hoc* effects, ^∗∗∗^*p* < 0.001 Gpm6a wt-EGFP vs K255A-EGFP, ^∗∗∗^*p* < 0.001Gpm6a wt-EGFP vs E258A-EGFP, *p* = 0.0551 Gpm6a wt-EGFP vs E259A-EGFP. **(B)** The mean fluorescence intensity of the surface-labeled Gpm6a measured by flow cytometry in the population of EGFP-positive N2a cells transfected with the indicated vectors. Surface Gpm6a was labeleld by immunostaining of non-permeabilized cells with the rat anti-Gpm6a antibody followed by goat anti-rat IgG conjugated to Alexa Fluor 647. Data are means ± SEM of three independent experiments. One-way ANOVA followed by Dunnett’s multiple comparisons test for *post hoc* effects, ^∗∗∗^*p* < 0.001 Gpm6a wt-EGFP vs K255A-EGFP, ^∗∗∗^*p* < 0.001 Gpm6a wt-EGFP vs E258A-EGFP.

The protein expression levels and the amount of Gpm6a present on cell surface were then quantified by flow cytometry in N2a cells transfected with the indicated EGFP-tagged mutants as described in the previous sections. For D253A-EGFP ∼13% (SEM ± 0.229) of the total cells assessed was EGFP-positive, for K255A-EGFP ∼8% (SEM ± 1.373), for E258A-EGFP ∼7% (SEM ± 0.3291), and for E259A-EGFP ∼9% (SEM ± 0.7318). This indicates that the transfection efficiency of D253A-EGFP did not differ from that of Gpm6a wt-EGFP (∼13%, SEM ± 0.623), while significantly less EGFP-positive cells were detected for K255A-EGFP, E258A-EGFP, and E259A-EGFP. The intensity of the fluorescence signal of EGFP as a measure of the amount of expressed protein was quantified in the fraction of EGFP-positive cells (**Figure 9A**). K255A and E258A displayed significantly lower level of EGFP fluorescence signal when compared to the Gpm6a wt-EGFP. No significant differences were observed for the mutant proteins D253A and E259A, although a tendency toward significance (*p* = 0.0551) was detected for E259A (**Figure 9A**). When the intensity of the fluorescence signal of the surface labeled Gpm6a was quantified in the fraction of EGFP-positive cells and compared to the wt Gpm6a, the difference was statistically significant for K255A and E258A displaying 19.6% and 29.3% lower amount of Gpm6a present on the cell surface, respectively (**Figure 9B**). These results indicate that the substitution of charged amino acids K255A and E258A with alanine interfere with the amount of expressed protein and with the amount of the Gpm6a protein on cell surface.

### Analysis of Gpm6a Amino Acid Sequence

**FIGURE 10:**
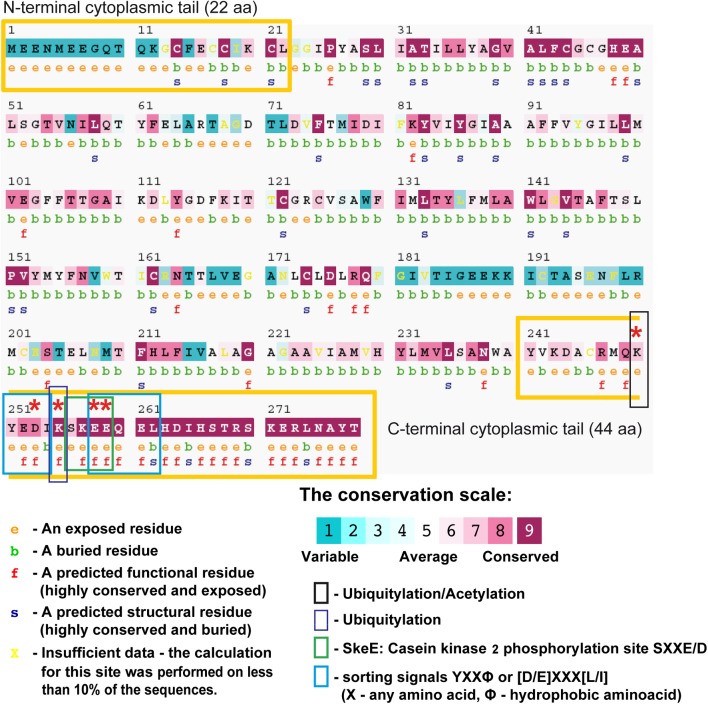
ConSurf analysis of the mouse Gpm6a (NCBI Accession: NP_705809.1) using 79 homologues obtained from the Ensembl database. The sequence of the query protein is displayed with the amino acids colored by their conservation grades using the color-coding bar (see legend). The residues of the query sequence are numbered starting from 1. The N-terminal and the C-terminal cytosolic domains are marked as yellow boxes according to the topological domain prediction based on UniProtKB database (**Supplementary Table S1**). The figure reveals that the C-terminal cytoplasmic tail is highly conserved and the N-terminal cytoplasmic tail is highly variable. The first row below the sequence lists the predicted burial status of the amino acid (“b”—buried vs “e”—exposed). The second row indicates residues predicted to be structurally and functionally important: “s” and “f,” respectively. Residues K250, D253, K255, E258, and E259 whose substitution with alanine lead to deficiencies in the formation of filopodia are indicated by asteriscs. Post-translational modifications or sequence motifs identified for these residues are indicated by colored boxes as shown in the legend (see **Tables 1**, **2** for the complete list of the post-translational modifications or sorting motifs identified in Gpm6a sequence).

The degree to which an amino acid position within a protein sequence is evolutionarily conserved is often indicative of its structural and functional importance. Conservation analysis of amino acid positions within Gpm6a was performed using a bioinformatics tool ConSurf (Berezin et al., 2004; Ashkenazy et al., 2010, 2016). ConSurf estimates the evolutionary conservation rate of amino acid residues in a protein molecule based on the phylogenetic relations between homologous sequences. Considering that the capacity of Gpm6a to form membrane protrusions is conserved among its orthologues (Alfonso et al., 2005; Huang et al., 2011; Zappia et al., 2012; Gregor et al., 2014), but not paralogues such as PLP (Fernandez et al., 2010), a multiple sequence alignment (MSA) of the Ensembl listed orthologues sequences of the mouse Gpm6a (NCBI Accession: NP_705809.1) was constructed using CLUSTALW algorithm. The MSA was then used by ConSurf to build a phylogenetic tree using the neighbor-joining algorithm with maximum likelihood (ML) distance. Position-specific conservation scores were computed using the Bayesian algorithm and the conservation scores were projected onto the protein sequence of the mouse Gpm6a (**Figure 10**). In the **Figure 10**, the conservation score at each position corresponds to the evolutionary rate of the residue. We observe that highly variable residues cluster mainly in three regions (topological domain prediction is based on UniProtKB database; **Supplementary Table S1**): the N-terminal cytoplasmic tail, the small extracellular loop EC1, and the large extracellular loop EC2 of Gmp6a. On the other hand, highly conserved residues cluster preferentially within the four transmembrane domains (TM1-4) and, most of all, within the C-terminal cytoplasmic tail (**Figure 10**).

In addition to estimated evolutionary rates, ConSurf assigns predicted relative solvent accessibility to each amino acid in the sequence. Both are subsequently used to indicate residues that have potential structural or functional importance. Functionally important residues that take part, for example in ligand binding and protein-protein interactions, are generally evolutionarily conserved and are most likely to be solvent-accessible, whereas conserved residues buried within the protein core have most probably an important structural role in maintaining the protein’s fold. We observe that the majority of predicted functional residues clusters in the C-terminal cytoplasmic tail. Among them D253, K255, E258, and E259 were identified as residues of potential functional importance. No predicted functional residues are detected in the N-terminal cytoplasmic tail.

Next, deletion of the C-terminal cytosolic domain or alanine substitutions may affect Gpm6a-induced filopodium formation by disrupting sites of post-translational modifications or sequence motifs that participate in this process. **Table 1** lists the post-translational modifications sites identified by various bioinformatics resources in the amino acid sequence of the mouse Gpm6a (NCBI Accession: NP_705809.1). K250 and K255 in the Gpm6a C-terminal are predicted as sites of ubiquitination by PhosphositePlus^®^ and E258 and E259 lie within a consensus sequence for casein kinase II (CK2) phosphorylation ^256^SKEE^259^ as predicted by PhosphositePlus^®^, UniProtKB and Prosite (**Table 1**). In addition, prediction of protein sorting signals by LOCATE database (Sprenger et al., 2008) identified various potential sorting signals in the amino acid sequence of the mouse Gpm6a (**Table 2**). Among them, ^251^YEDI^254^ and ^258^EEQEL^262^ are localized within the C-terminal cytosolic tail of Gpm6a and conform to the YXXØ consensus motif of tyrosine-based sorting signals and to the [D/E]XXX[L/I] motif of leucine-based sorting signal, respectively (McMahon and Mills, 2004). D253 lies within the ^251^YEDI^254^ sequence and E258 and E259 lie within the ^258^EEQEL^262^ sequence.

**Table 1:** Post-translational modification sites in Gpm6a (mouse Gpm6a NCBI Reference Sequence NP_705809.1; ^∗^Acetylation in K250 and K257 is listed only in rat ortholog NP_835206.1).

Site	Modification	Bioinformatics resource
T10	Phosphorylation *TqK*: PKC phospho site	Prosite
13–18	*Myristoylation* GCfeCC	Prosite
C17	*Palmitoylation*	SwissPalm
24–29	*Myristoylation* GIpyAS	Prosite
T60	Phosphorylation *TyfE*: CK2 phospho site	Prosite
T67	Phosphorylation *TagD*: CK2 phospho site	Prosite
T76	Phosphorylation *TmiD*: CK2 phospho site	Prosite
87–92	*Myristoylation* GIaaAF	Prosite
C122	*Palmitoylation*	SwissPalm
N164	N-glycosylation	UniProtKB/Prosite
T166	Phosphorylation *TlvE*: CK2 phospho site	Prosite
170–175	*Myristoylation* GAnlCL	Prosite
C174–C192	Disulfide bond	UniProtKB
T184	Phosphorylation *TigE*: CK2 phospho site	Prosite
T193	Phosphorylation *TasE*: CK2 phospho site	Prosite
C202	*Palmitoylation*	SwissPalm
N208	N-glycosylation	UniProtKB/Prosite
220–225	*Myristoylation* GAgaAV	Prosite
**K250**	Ubiquitylation/Acetylation^∗^	PhosphoSitePlus^®^
Y251	Phosphorylation	PhosphoSitePlus^®^
**K255**	Ubiquitylation	PhosphoSitePlus^®^
**S256**	Phosphorylation *SkeE*: CK2 phospho site	PhosphoSitePlus^®^/UniProtKB/Prosite
K257	Ubiquitylation/Acetylation^∗^	PhosphoSitePlus^®^
S267	Phosphorylation *StR*: PKC phospho site	PhosphoSitePlus^®^/Prosite
T268	Phosphorylation	PhosphoSitePlus^®^
S270	Phosphorylation	PhosphoSitePlus^®^
T278	Phosphorylation	PhosphoSitePlus^®^/UniProtKB


**Table 2:** Sorting motifs in mouse Gpm6a (NCBI Reference Sequence NP_705809.1) identified by subcellular localization database LOCATE (McMahon and Mills, 2004; Sprenger et al., 2008).

Motif	Function	Position/Range
YXXΦ	signals TGN-endosome sorting, plasma membrane exocytosis, melanosome biogenesis, basolateral sorting	61–64 114–117 153–156 **251–254**
[D/E]XXX[L/I]	signals TGN-endosome sorting, melanosome biogenesis	169–173 187–191 203–207 **258–262**


*X = any amino acid, Φ = hydrophobic amino acid.*

### Deletion of the C-Terminal Cytosolic Domain Diminishes Colocalization of Gpm6a With Clathrin

Motifs identified by bioinformatics resources in the previous section are described as being involved in recognition of cargo by accessory proteins in clathrin mediated trafficking events (McMahon and Mills, 2004). In addition, ubiquitination of cytosolic lysine residues also serves as a signal for sorting of transmembrane proteins in a manner that is dependent on clathrin (Bonifacino and Traub, 2003). We have previously identified clathrin heavy chain to coimmunoprecipitate with Gpm6a in rat hippocampal neurons (Fuchsova et al., 2015). Moreover, the colocalization of Gpm6a with clathrin in Gpm6a-overexpressing neurons and Hek293 cells was recently observed by Garcia and coworkers, who suggested that Gpm6a endocytic/recycling pathway involves clathrin (Garcia et al., 2017).

**FIGURE 11:**
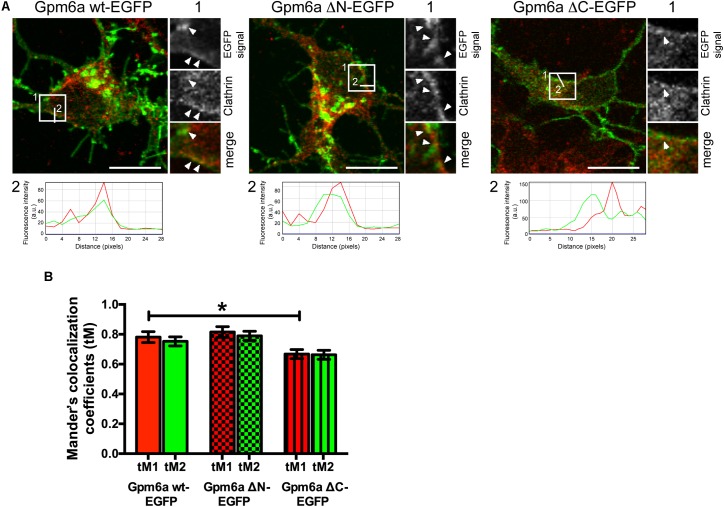
Deletion of the C-terminal cytosolic domain diminishes colocalization of Gpm6a with clathrin in hippocampal neurons. **(A)** Confocal images of hippocampal neurons (4 DIV) transfected with the indicated vectors (green) and immunostained with antibody against clathrin (red). A portion of Gpm6a-labeled spots colocalizes with clathrin upon overexpression of Gpm6a wt-EGFP and Gpm6a ΔN-EGFP (arrowheads; insets 1). Colocalization diminishes upon overexpression of Gpm6a ΔC-EGFP (arrowhead; inset 1). Fluorescence intensity profiles of the Gpm6a wt, or the ΔN-, or the ΔC- (green) and the anti-clathrin (red) along the white lines 2 indicated in the corresponding ROIs show the overlap of both signals. Scale bar, 20 μm. **(B)** Colocalization was evaluated in ROIs (25 × 25 pixels) as described in the Methods section. Mander’s colocalization coefficients using the calculated thresholds (tM) were determined for the red and the green channel. Ten to twenty neurons per group done in duplicates were analyzed. Data are means ± SEM of two independent experiments. One-way ANOVA followed by Dunnett’s multiple comparison test for *post hoc* effects. ^∗^*p* < 0.05 tM1 Gpm6a wt-EGFP vs tM1 Gpm6a ΔC-EGFP, *p* = 0.082 tM2 Gpm6a wt-EGFP vs tM2 Gpm6a ΔC-EGFP.

To evaluate whether clathrin mediated trafficking events can be affected by deletion of the N- or C-terminal cytosolic domain, we assessed the colocalization of the mutant proteins with clathrin in primary hippocampal neurons. Neurons of 3 DIV were transfected with the indicated EGFP-tagged mutants, immunostained with the antibody against clathrin, and analyzed by confocal microscopy (**Figure 11**). Overexpression of Gpm6a wt-EGFP was used as a control. Consistent with previous findings (Garcia et al., 2017), we observed that some of the clathrin-labeled spots were associated with the wt Gpm6a (**Figure 11A**, maximized view 1, arrowheads). The profile plot shows the overlap of the Gpm6a wt-EGFP (green) and anti-clathrin (red) fluorescence intensity peaks along a straight line 2 as indicated in the micrograph (**Figure 11A**, white line 2). Similar staining pattern was observed for Gpm6a ΔN-EGFP while Gpm6a ΔC-EGFP displayed more evenly dispersed cytosolic localization (**Figure 11A**). For quantification purposes, colocalization analysis of confocal images was performed using the Colocalization Analysis plugins of ImageJ. Mander’s colocalization coefficients using the calculated thresholds (tM) were determined for the analyzed regions of interest (ROIs 25 × 25 pixels). For clathrin and Gpm6a wt-EGFP, tMs were 0.781 (SEM ± 0.03676) for the red channel (tM1) and 0.7526 (SEM ± 0.03054) for the green channel (tM2). For clathrin and Gpm6a ΔN-EGFP: tM1 = 0.8151 (SEM ± 0.03644) and tM2 = 0.7899 (SEM ± 0.03074); for clathrin and Gpm6a ΔC-EGFP: tM1 = 0.6678 (SEM ± 0.03050) and tM2 = 0.6625 (SEM ± 0.03028). When compared to the control wt Gpm6a, decrease in Mander’s colocalization coefficients of the Gpm6a ΔC-EGFP was statistically significant for the red channel and displayed tendency toward significance for the green channel (*p* = 0.082). No statistically significant difference was observed for the Gpm6a ΔN-EGFP (**Figure 11B**). Taken together, our colocalization assays in hippocampal neurons suggest that deletion of the C-terminal, but not the N-terminal, cytosolic domain diminishes the association of Gpm6a with clathrin implying involvement of clathrin mediated trafficking events.

## Discussion

Filopodia perform fundamental roles in neuronal development and circuit formation including initiation, outgrowth and guidance of axons and dendrites, generation of axon collateral and dendrite branches, and formation of synaptic structures (Gallo, 2013). Neuronal glycoprotein Gpm6a functions in the processes of neural development such as outgrowth of neurites, differentiation, and synaptogenesis. At the same time, this four-transmembrane-domain protein is a potent inducer of filopodia irrespective of cell type or subcellular compartment in which it is expressed (Lagenaur et al., 1992; Mukobata et al., 2002; Alfonso et al., 2005; Michibata et al., 2008; Zhao et al., 2008; Fuchsova et al., 2009; Brocco et al., 2010; Huang et al., 2011; Scorticati et al., 2011; Formoso et al., 2015; Mita et al., 2015; Honda et al., 2017; Ito et al., 2018). In structurally similar tetraspanins, functional specificity is determined by the second large extracellular loop EC2 and the cytoplasmic tails function as connections with cytoskeletal or signaling proteins (Hemler, 2005; Charrin et al., 2009; Yanez-Mo et al., 2009). The functional importance of the Gpm6a large extracellular loop EC2 in the process of filopodium formation has already been demonstrated (Fuchsova et al., 2009), however, the cytosolic N- and C-terminal tails have received less attention.

In the present work, we map the regulatory effect of Gpm6a in filopodium formation to its C-terminal cytoplasmic region. We observe that deletion of the C-terminal (Δ243–278) cytosolic end, but not the N-terminal (Δ1–16), prevents formation of filopodia triggered by Gpm6a in N2a cells and in cultured neurons from the rat hippocampus. In accordance with this observation, we determine using the bioinformatic tool ConSurf that the Gpm6a C-terminus contains amino acids residues that are highly conserved across several animal species suggesting functional importance, while the N-terminal cytoplasmic tail is highly variable. ConSurf also determines that the majority of predicted functional residues (evolutionarily conserved and exposed) map to the C-terminal cytoplasmic tail, while no predicted functional residues are detected in the N-terminal. In line with the fact that the capacity of Gpm6a to form filopodia is conserved among its orthologues (Alfonso et al., 2005; Huang et al., 2011; Zappia et al., 2012; Gregor et al., 2014), our results indicate that the function of Gpm6a in filopodium formation is linked to its highly conserved C-terminus. The functional importance of the Gpm6a C-terminus is consistent with a number of data highlighting the essential role of the C-terminal tail in the function and molecular organization of other tetraspanins. For example, TSPAN7 interacts with its C-terminal end with protein interacting with C kinase 1 (PICK1), to regulate PICK1 and GluR2/3 association and AMPA receptor trafficking. Overexpression of full length TSPAN7, but not the mutant lacking C terminus, induces filopodial outgrowth in COS7 cells as well as in hippocampal neurons (Bassani et al., 2012). Next, the C-terminal cytoplasmic domain of the tetraspanin CD63 interacts with syntenin-1, a new regulator of endocytosis that can counteract internalization of CD63 (Latysheva et al., 2006). Another tetraspanin CD81 regulates cell migration via the interaction of its C-terminal cytosolic domain with Rac GTPase (Tejera et al., 2013).

Interestingly, Sato and coworkers have demonstrated that the N-terminal intracellular domain of Gpm6a (aa 1–25) plays a role in the axon outgrowth arrest without growth cone collapse and the C-terminal intracellular domain (aa 238–278) was shown to be dispensable for this process (Sato et al., 2011). We can speculate that different cytoplasmic domains of Gpm6a could participate in different neuroplastic events such as axonal growth or formation of dendritic filopodia, possibly through the specific binding of these domains with different intracellular proteins.

Proper EC2 folding and TM domain assembly is required for surface expression of PLP/DM20, a tetraspan protein from the same family of proteolipid proteins as Gpm6a. It has been suggested that “the alignment of TM domains organizes the overall topology of a polytopic membrane protein and that the freedom to realign TM domains determines the impact of mutations in extracellular domain. If the tetraspan itself cannot form because of truncations, frame-shift, or missense mutations affecting the TM domains, PLP/DM20 is retained in the endoplasmatic reticulum (ER)” (Dhaunchak et al., 2011). Here, we show that deletion of the N- or the C-terminal intracellular domains of Gpm6a does not prevent the recognition of Gpm6a by a function blocking anti-Gpm6a antibody directed against EC2 domain suggesting that the conformation of EC2 is not altered. Nevertheless, we could observe that the amount of surface Gpm6a is lower in both cases. This leads us to hypothesize that proper protein folding and/or cell surface trafficking could be partially affected by removing both the N- and the C-terminal intracellular regions but the formation of filopodia is mediated only by the C-terminus.

The subsequent charged-to-alanine scanning mutagenesis of the C-terminal end allowed us to identify residues that are functionally critical for the process of filopodia formation. We observed that the substitution with alanine of charged amino acids K250, D253/K255, and E258/E259 prevented formation of filopodia in N2a cells and in cultured neurons from rat hippocampus. When separate substitution with alanine of charged amino acids D253, K255, E258, and E259 was done, the only point mutation that interfered with filopodium outgrowth in both N2a and in primary hippocampal neurons was E258. For K255, the effect was observed only in hippocampal neurons. Point mutation of D253 and E259 did not lead to inhibition of filopodium formation.

One possibility is that, similarly to tetraspanins, the functionally critical residues identified in our study could participate in the interaction of the C-terminal end of Gpm6a with other proteins that can directly regulate actin cytoskeleton dynamics or cell signaling, and by this manner facilitate the formation of filopodia. The interaction would be disrupted by mutating the residues. The rationale behind this hypothesis is that the charged amino acids are more probably to be located on the surface of the protein and thus more probably to paricipate in interactions with other proteins (Gibbs and Zoller, 1991). Indeed, in line with this notion, amino acid residues K255 and E258 were identified as predicted functional residues (evolutionarily conserved and exposed) by ConSurf analysis. Regarding possible interacting partners, in our recent work we have shown that the actin regulator Coronin 1a colocalizes and coimmunoprecipitates with Gpm6a in rat hippocampal neurons and that the outgrowth of filopodia triggered by Gpm6a is facilitated by Coronin 1a and Rac1/Pak1 signaling pathway (Fuchsova et al., 2015; Alvarez Julia et al., 2016). Moreover, Rac1 was shown to coimmunoprecipitate with Coronin 1a together with Gpm6a (Alvarez Julia et al., 2016). In addition, clathrin heavy chain and other proteins were identified to coimmunoprecipitate with Gpm6a in rat hippocampal neurons (Fuchsova et al., 2015). We could speculate that the residues K255 and E258 participate in the interaction of the C-terminal end of Gpm6a with Coronin 1a or other proteins (such as Rac1 or clathrin) and this interaction is necessary for the formation of filopodia by Gpm6a. In accordance with our hypothesis, follow up experiments were performed and showed that the recombinant GST-fused C-terminal end of Gpm6a of 30 amino acids coimunoprecipitates with Coronin 1a using anti-coronin 1a antibody (**Supplementary Figure S4**). Although additional future work is required to further characterize the interaction, these preliminary data prove the relevance of our findings.

Alternatively, it is possible that by mutating the residues we identified as functionally critical post-translational modifications or structural motifs that participate in the filopodium formation process are lost. Analysis of Gpm6a sequence revealed various signaling motifs that would be disturbed by alanine substitution of these residues. First, K250 and K255 are predicted as sites of ubiquitination. “Ubiquitination is a reversible post-translational modification that regulates a multitude of physiological processes, including protein degradation, endocytosis and the sorting and trafficking of transmembrane proteins” (Hershko and Ciechanover, 1998). In neurons, this pathway plays multiple roles and has been described as an emergent mechanism for regulating synapse function and plasticity (Mabb and Ehlers, 2010). For example, ubiquitination of AMPA receptors regulates the intracellular sorting of receptors to late endosomes for degradation in lysosomes (Widagdo et al., 2017).

Second, E258 forms a part of the ^258^EEQEL^262^ sequence that conform to the [D/E]XXX[L/I] motif of leucine-based sorting signal according to the LOCATE database (McMahon and Mills, 2004; Sprenger et al., 2008). The original consensus motif for a mono-leucine sorting signal consists of a single leucine five residues C-terminal to an acidic cluster (EEDXXXXXL) and it is present in two other proteins, CD147 and stem cell factor (SCF) (Wehrle-Haller and Imhof, 2001; Deora et al., 2004). In addition, a variation of this consensus mono-leucine sorting motif (EEXXXL) was identified within the cytoplasmic domain of amphiregulin where it regulates biosynthetic delivery of amphiregulin to the basolateral surface (Gephart et al., 2011). Although Gpm6a does not contain this exact consensus motif, it does contain a mono-leucine C-terminal to an acidic cluster (EEXXL).

Canonical di-leucine sorting motif is not present in the C-terminal cytosolic end of Gpm6a but there is the ^251^YEDI^254^ sequence that conforms to the YXXØ consensus motif of tyrosine-based sorting signals. The substitution of E252 or D253 with alanine in our study did not affect filopodium formation implying the notion that ^251^YEDI^254^ motif is dispensable for the process of filopodium formation. Accordingly, previous work by Formoso and coworkers showed that the replacement of the tyrosine residue at position 251 by alanine affects only neurite extension but not filopodium formation (Formoso et al., 2015). On the other hand, the Y251A mutation was shown to totally abolish Gpm6a internalization induced by the monoclonal antibody without interfering in its immunodetection which led to the conclusion that Gpm6a endocytosis is mediated through the ^251^YEDI^254^ motif (Garcia et al., 2017).

Published works on sorting of plasma membrane proteins demostrated that there are proteins such as transferrin receptor(TfR) that use different sorting signals at trans Golgi network (TGN) and endosomes during biosynthetic delivery and post-endocytic recycling to the plasma membrane (Odorizzi and Trowbridge, 1997). “Mutations have been identified that selectively impair basolateral sorting of internalized TfRs from the endocytic pathway without affecting basolateral sorting of newly synthesized receptors implying that there are subtle differences in the recognition of the TfR basolateral sorting signal by separate sorting machinery located within the biosynthetic and endocytic pathways” (Odorizzi and Trowbridge, 1997). In this context, we can speculate that Gpm6a trafficking may use different motifs and different adaptors at each location in biosynthetic or recycling pathway.

E258 also lies within a consensus sequence for CK2 phosphorylation ^256^SKEE^259^. It is of interest to note that S256 was indeed identified as a site of phosphorylation by the phosphoproteome analysis of postmortem Alzheimer’s disease brain tissue (Xia et al., 2008) and phosphorylation of the zebrafish ortholog M6Ab at serine 263, which corresponds to serine 256 of mouse Gpm6a, was shown to contribute to filopodium formation in PC12 cells and neurite outgrowth in zebrafish embryos (Huang et al., 2011). On the other hand, in rat hippocampal neurons, overexpression of Gpm6a bearing simultaneous mutations of various putative intracellular phosphorylation sites (including S256) did not affect formation of filopodia but did lower filopodium motility. Nevertheless, the effect of mutation of individual intracellular phosphorylation sites was not addressed by this study (Brocco et al., 2010). It is of note that “sorting motifs that consists of clusters of acidic residues containing sites for phosphorylation by CK2 are often found in transmembrane proteins and play a role in retrieval from endosomes to the TGN” (Bonifacino and Traub, 2003). A monomeric protein named PACS-1 (phosphofurin acidic cluster sorting protein 1) was identified that binds to acidic clusters in a CK2 phosphorylation dependent manner and functions as a connector that links the phosphorylated acidic clusters to the clathrin-dependent sorting machinery (Wan et al., 1998; Crump et al., 2001).

Taken together, using alanine scanning mutagenesis we identified amino acids in the C-terminal cytosolic end of Gpm6a essential in the process of filopodium formation that are predicted as parts of sorting motifs. In this context, diminished surface expression of mutant proteins we observe in our study could indicate that Gpm6a trafficking is disturbed by replacement of these residues. On the other hand, decreased total protein expression of mutant proteins K255A and E258A suggests their degradation probably due to the destabilizing effect on protein folding. Future work is required to establish whether and which biosynthetic or internalization pathways are affected by individual mutations.

Membrane traffic systems in non-neuronal cells contribute to cell morphogenesis and appear to be driving factors for cell polarization. In neurons, filopodial processes were identified as one of the hot spots of active membrane remodeling where endocytic membrane retrieval initiates in the growth cone during axon extension (Hines et al., 2012). Moreover, membrane trafficking from recycling endosomes is required for the growth and maintenance of spines and regulated membrane trafficking of postsynaptic neurotransmitter receptors has emerged as a central mechanism for synapse development and modification (Ehlers, 2000; Park et al., 2004, 2006). Consistently, Gpm6a has been suggested to facilitate micro-opioid receptor (and a number of other GPCRs) endocytosis and recycling (Wu et al., 2007; Liang et al., 2008) and endocytic sorting and recycling of Gpm6a has been shown to affect neuronal synapses (Garcia et al., 2017). Our observation that deletion of the C-terminal, but not the N-terminal, cytosolic domain diminishes colocalization of Gpm6a with clathrin further points to the functional significance of the C-terminal end of Gpm6a and to the involvement of clathrin mediated trafficking events in the process of filopodium formation induced by Gpm6a.

## Author Contributions

BF conceived and designed the study. BF, NR, and AAJ performed the experiments. BF, NR, AAJ, and SA analyzed the data. BF, NR, AAJ, and AF participated in the interpretation of data. BF wrote the paper. NR wrote sections of the manuscript. All authors contributed to manuscript revision, read and approved the submitted version.

## Conflict of Interest Statement

The authors declare that the research was conducted in the absence of any commercial or financial relationships that could be construed as a potential conflict of interest.

## Abbreviations

EGFP: enhanced green fluorescent protein
Gpm6a: neuronal membrane glycoprotein M6a
N2a: mouse neuro-2a neuroblastoma cell line
ROI: region of interest
TBS: tris-buffered saline
