# Supplementary material for: Alanine Scanning Mutagenesis of the C-Terminal Cytosolic End of Gpm6a Identifies Key Residues Essential for the Formation of Filopodia
Source: Front Mol Neurosci. 2018 Sep 4;11:314. doi: 10.3389/fnmol.2018.00314 (PMC6131581; doi:10.3389/fnmol.2018.00314)
Supplement: Supplementary file 1 [file Data_Sheet_1.pdf]

Supplementary Material

Alanine Scanning Mutagenesis of the C-Terminal Cytosolic End of Gpm6a Identifies Key Residues Essential for the Formation of Filopodia

Nicolás Matías Rosas[‡], Anabel Alvarez Juliá[‡], Sofia Elisa Alzuri, Alberto Carlos Frasch, and Beata Fuchsova*

[‡]equal contribution

*** Correspondence:** Beata Fuchsova: beata@iibintech.com.ar

1 Supplementary Table and Figures

1.1 Supplementary Table

Feature key	Position (amino acid)	Description	Length
Topological domain	1-22	Cytoplasmic/ N-terminus	22
Transmembrane	23-43	Helical/ TM1	21
Topological domain	44-84	Extracellular/ EC1	41
Transmembrane	85-105	Helical/ TM2	21
Topological domain	106-127	Cytoplasmic/ IC	22
Transmembrane	128-148	Helical/ TM3	21
Topological domain	149-213	Extracellular/ EC2	65
Transmembrane	214-234	Helical/ TM4	21
Topological domain	235-278	Cytoplasmic/ C-terminus	44

Supplementary Table S1: Predicted topology of Gpm6a (UniProtKB - P35802 GPM6A_MOUSE): four transmembrane domains (TM1-4), two extracellular loops (EC1 and EC2), small intracellular loop (IC), and the N- and C-terminal regions facing the cytoplasm.

1.2 Supplementary Figures

```
Gpm6a  ENSMUSP00000033915  KDA----------CRMQKYEDIKSKEEQELHDIHSTRSKERLNAYT
Plp    ENSMUSP00000033801  ----------------------------------------------
Plp    ENSMUSP00000033800  KDALMGRGTKF-----------------------------------
Plp    ENSMUSP00000108708  KDALMGRGTKF-----------------------------------
Gpm6b  ENSMUSP00000107847  K----------DASKMQAYQDIKAKEEQELQDIQSRSKEQLNSYT-
Gpm6b  ENSMUSP00000107852  K----------FKSR----EDCCTKF--------------------
Gpm6b  ENSMUSP00000107846  K----------FKSR----EDCCTKF--------------------
Gpm6b  ENSMUSP00000107845  K----------FKSR----EDCCTKF--------------------
Gpm6b  ENSMUSP00000107855  K----------DASKMQAYQDIKAKEEQELQDIQSRSKEQLNSYT-
Gpm6b  ENSMUSP00000107848  K----------DASKMQAYQDIKAKEEQELQDIQSRSKEQLNSYT-
Gpm6b  ENSMUSP00000107842  K----------FKSR----EDCCTKF--------------------
Gpm6b  ENSMUSP00000107854  K----------FKSR----EDCCTKF--------------------
Gpm6b  ENSMUSP00000107843  K----------FKSR----EDCCTKF--------------------
Gpm6b  ENSMUSP00000060442  K----------DASKMQAYQDIKSKEEQELHDIHSTRSKERLNAYT
```

Supplementary Figure S1: Charged residues identified as conserved in homologous proteins from the same family. Multiple sequence alignment of the C-terminal end (aa 243-278) of the mouse Gpm6a with sequences of the members of the proteolipid protein (PLP) family, Plp and Gpm6b, constructed in Ensembl using CLUSTALW algorithm. Individual isoforms ID numbers are listed according to Ensembl. Charged amino acids identified as universally highly conserved are highlighted in red.

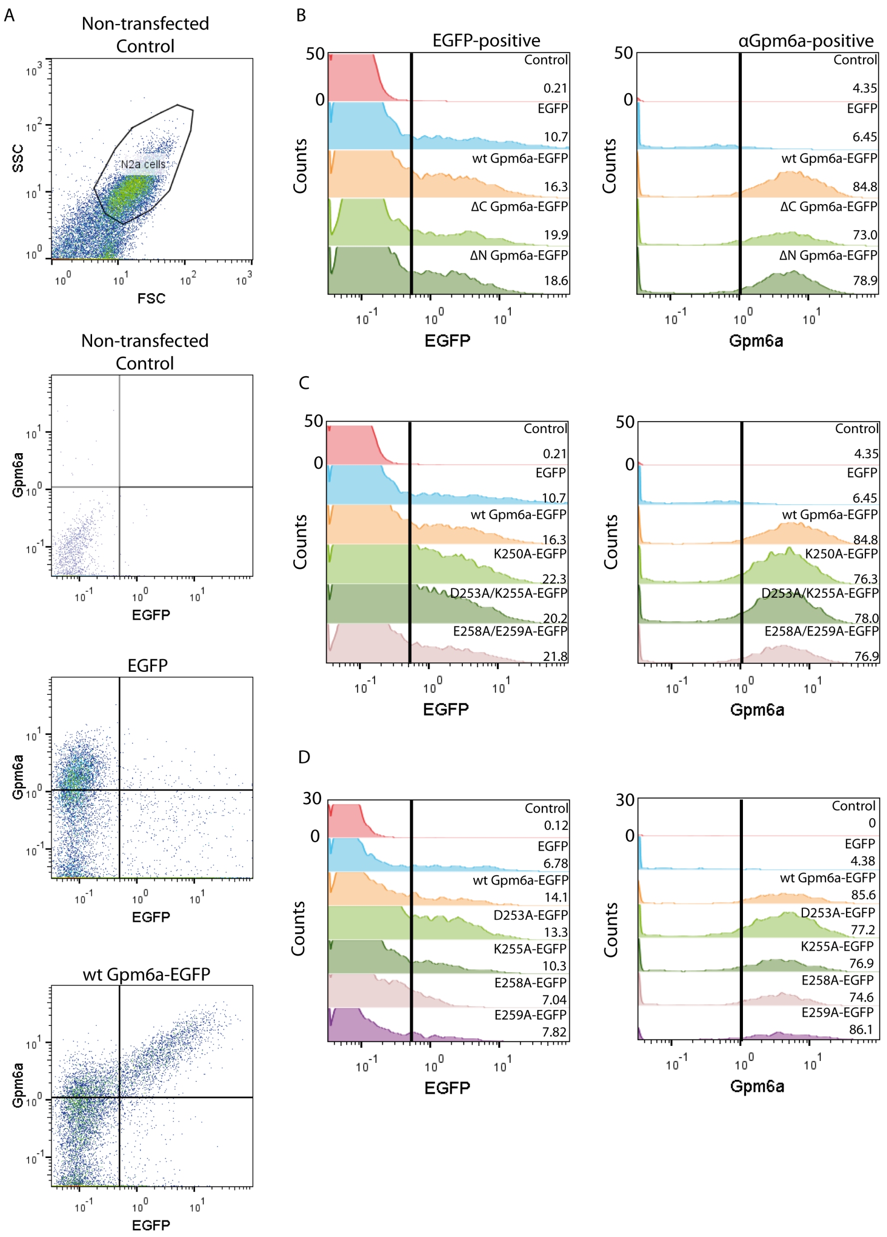

Supplementary Figure S2: Quantification of the protein expression levels and the amount of Gpm6a on cell surface in N2a cells using flow cytometry. N2a transfected with the indicated EGFP-tagged mutants were fixed and Gpm6a present on cell surface was labeled by immunostaining of non-permeabilized cells with the rat anti-Gpm6a mAb. EGFP-tagged wt Gpm6a, EGFP alone and non-transfected cells were used as controls. Examples of representative plots from individual measurements are shown. For quantification purposes two or three independent experiments were performed for each transfection condition and measurements were done in triplicates. (A) Gating of N2a cells population using a side scatter (SSC) and forward scatter (FSC) criteria. The fluorescence intensity of the non-transfected intact cells immunostained only with the secondary antibody, as well as the intensity of the cells transfected with EGFP alone and EGFP-tagged wt Gpm6a stained with both the primary and the secondary antibody were used as controls to define EGFP and Gpm6a positive and negative N2a populations. (B, C, D) Gating of EGFP-positive N2a cells transfected with the indicated vectors,

left. Gating of surface Gpm6a-positive cells within the population of EGFP-positive cells transfected with the indicated vectors, right. The numbers indicated refer to the percentage of positive counts for the individual measurement shown.

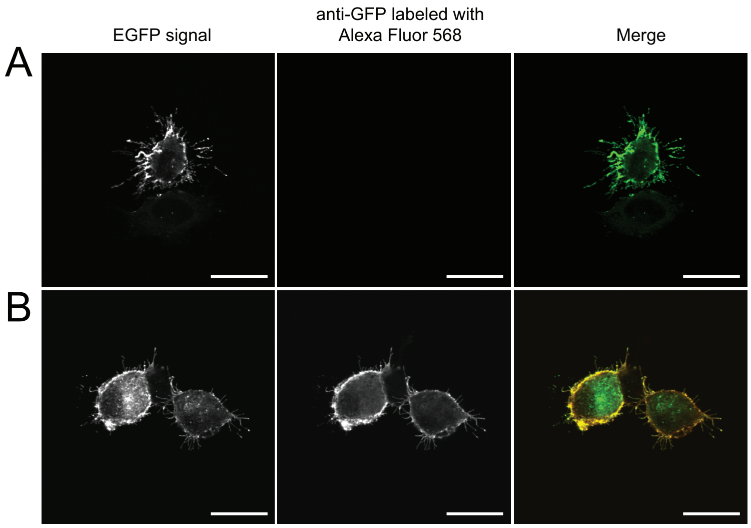

Supplementary Figure S3: Intracellular antigen detection in non permeabilized (A) or permeabilized (B) N2a cells. Confocal images of N2a cells transfected with Gpm6a wt-EGFP and labeled with the rabbit anti-GFP antibody followed by the goat anti-rabbit IgG conjugated to Alexa Fluor 568. (A) Living non permeabilized N2a cells transfected with Gpm6a wt-EGFP were immunostained on ice. Cells were subsequently fixed with paraformaldehyde. No signal is detected in the red channel indicating that the antibody does not pass the plasma membrane and does not recognize EGFP tag of Gpm6a localized on the cytosolic side of the plasma membrane. (B) Cells permeabilized and fixed before the labeling. Anti-GFP signal is readily detected in the red channel Scale bar, 10 μm.

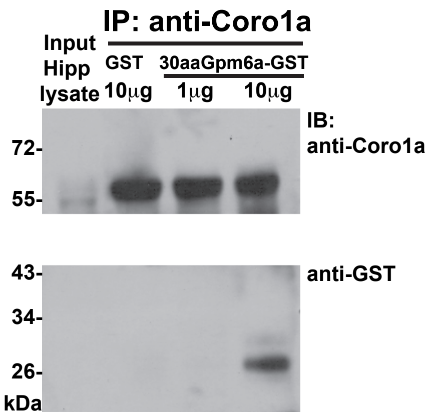

Supplementary Figure S4: GST-fused C-terminal end of Gpm6a of 30 amino acids coimmunoprecipitates with Coronin 1a using the anti-coronin 1a antibody. Rat hippocampal lysates were previously incubated with GST-fused 30 amino acids C-terminal peptide of Gpm6a. Incubation with GST alone was used as a control. Protein extracts were subsequently incubated with the rabbit anti-Coro1a antibody and immune complexes were collected by binding to the Protein G-Sepharose beads. Immunoprecipitation (IP) was performed as described in Alvarez Julia et al. 2016. Coro1a-coimmunoprecipitating proteins were subsequently analyzed by SDS-PAGE followed by Western blot. Immunoblots (IB) were analyzed with the anti-Coro1a antibody and anti-GST antibody.

Alvarez Julia, A., Frasch, A.C., and Fuchsova, B. (2016). Neuronal filopodium formation induced by the membrane glycoprotein M6a (Gpm6a) is facilitated by coronin-1a, Rac1, and p21-activated kinase 1 (Pak1). *J Neurochem* 137, 46-61.
